# Biological significance of soluble IL-2 receptor

**DOI:** 10.1155/S0962935193000018

**Published:** 1993

**Authors:** Calogero Caruso, Giuseppina Candore, Diego Cigna, Antonio Tobia Colucci, Maria Assunta Modica

**Affiliations:** Istituto di Patologia generale Universita' di Palermo Corso Tukory 211 Palermo 90134 Italy

## Abstract

A NUMBER of receptors for growth factors and differentiation antigens have been found to be secreted or released by cells. Following mononuclear cell (MNC) activation and interleukin-2 receptor (IL-2R) expression, a soluble form of the Alpha;-chain of IL-2R (sIL-2R) is released. The sIL-2R has been shown to be present in the culture supernatants of activated MNCs as well as in normal sera and, in higher amounts, in sera from subjects affected by several diseases including neoplastic, infectious and autoimmune ones, and in sera from transplanted patients suffering allograft rejection. The blood sIL-2R levels depend on the number of producing cells and the number of molecules per cell, so that sIL-2R blood values may represent an index of the number and the functional state of producing cells, both normal and neoplastic. Thus, monitoring of the immune system, mostly T-cells and haematological malignancies might be targets for the measurement of sIL-2R. Since many conditions may influence sIL-2R production, little diagnostic use may result from these measurements. However, since blood sIL-2R levels may correlate with disease progression and/or response to therapy, their measurement may be a useful index of activity and extent of disease. The precise biological role of the soluble form of the IL-2R is still a matter of debate. However, we know that increased sIL-2R levels may be observed in association with several immunological abnormalities and that sIL-2R is able to bind IL-2. It is conceivable then that in these conditions the excess sIL-2R released *in vivo* by activated lymphoid cells or by neoplastic cells may somehow regulate IL-2-dependent processes. On the other hand, it cannot exclude that sIL-2R is a by-product without biological significance. Finally, it is puzzling that in many conditions in which an increase of blood sIL-2R values has been observed, MNCs display a decreased *in vitro* capacity to produce sIL-2R. These seemingly contrasting findings are discussed in the light of the data showing that sIL-2R production correlates with IL-2 production.


Mediators of Inflammation, 2, 3-21 (1993)

### Biological significance of soluble IL-2 receptor

Calogero Caruso[CA], Giuseppina Candore, Diego Cigna, Antonio Tobia Colucci and Maria Assunta Modica

Istituto di Patologia generale, Universita' di Palermo, Corso Tukory 211, 90134 Palermo, Italy

[CA] Corresponding Author

**A NUMBER of receptors for growth factors and differentiation antigens have been found to be secreted or released by cells. Following mononuclear cell (MNC) activation and interleukin-2 receptor (IL-2R) expression, a soluble form of the α-chain of IL-2R (sIL-2R) is released. The sIL-2R has been shown to be present in the culture supernatants of activated MNCs as well as in normal sera and, in higher amounts, in sera from subjects affected by several diseases including neoplastic, infectious and autoimmune ones, and in sera from transplanted patients suffering allograft rejection. The blood sIL-2R levels depend on the number of producing cells and the number of molecules per cell, so that sIL-2R blood values may represent an index of the number and the functional state of producing cells, both normal and neoplastic. Thus, monitoring of the immune system, mostly T-cells and haematological malignancies might be targets for the measurement of sIL-2R. Since many conditions may influence sIL-2R production, little diagnostic use may result from these measurements. However, since blood sIL-2R levels may correlate with disease progression and/or response to therapy, their measurement may be a useful index of activity and extent of disease. The precise biological role of the soluble form of the IL-2R is still a matter of debate. However, we know that increased sIL-2R levels may be observed in association with several immunological abnormalities and that sIL-2R is able to bind IL-2. It is conceivable then that in these conditions the excess sIL-2R released *in vivo* by activated lymphoid cells or by neoplastic cells may somehow regulate IL-2-dependent processes. On the other hand, it cannot exclude that sIL-2R is a by-product without biological significance. Finally, it is puzzling that in many conditions in which an increase of blood sIL-2R values has been observed, MNCs display a decreased *in vitro* capacity to produce sIL-2R. These seemingly contrasting findings are discussed in the light of the data showing that sIL-2R production correlates with IL-2 production.**

**Key words:** Activation, Autoimmunity, B-cell, IL-2, IL-2R, Malignancies, Monocyte, sIL-2R, T-cell

### Interleukin-2

In 1976, the presence of a T-cell growth promoting activity in the supernatants of activated T-cell cultures was reported. Many laboratories have since contributed to the characterization of this lymphokine, now designated IL-2. IL-2 is the most well-defined and characterized interleukin because of its pivotal role in the generation of immune response and because of its biological property of maintenance of T-cell proliferation *in vitro*, which has resulted in considerable effort being devoted to its purification and characterization. IL-2 induces T-cell proliferation in an autocrine and paracrine manner and provides a means by which T-cells can be clonally expanded *in vitro*. The nucleotide sequence encoding IL-2, the genomic structure and the amino acid sequence giving rise to IL-2 activity have all been established. IL-2 is a single peptide of 15.5 kDa, encoded by a gene on the long arm of chromosome 4, produced by T-cells (most CD4, but also CD8) and natural killer (NK) cells. *In vitro* IL-2 synthesis is induced by a variety of stimuli, besides specific antigens, including antibodies reacting with cell surface molecules involved in activation pathways and nonspecific activating substances such as lectins. The activity of IL-2 is not confined to T-cells; it can act as a growth and differentiation factor for B-cells and NK cells and can activate macrophages. In transgenic mice constitutively producing high levels of IL-2, the major effect appears to be on the production of NK cells.[1-4]

Recent advances, derived from studies in the mouse, have demonstrated that functional subsets of cells with otherwise indistinguishable surface

© 1993 Rapid Communications of Oxford Ltd phenotypes can be defined by the patterns of cytokines produced. That has allowed the delineation of two subsets of CD4+ T-cells in the mouse: T-helper (Th1)-cells, involved in delayed type hypersensitivity (DTH), which secrete IL-2 and interferon-gamma (IFN-τ) and Th2-cells, involved in B-cell activation, which produce IL-4, IL-5 and IL-6, but not IL-2 and IFN-τ. Recent reports from several laboratories studying T-cell clones from individuals whose immune systems are actively engaged by antigens (e.g. subjects affected by allergies or infectious diseases), suggest that functional subsets of CD4+ T-cells exist also in man. This analysis has been extended to CD8+ T-cell clones which can similarly be divided into functional subsets based on the patterns of cytokine production.[5–7]

In peripheral T-cells, IL-2 induction depends on a series of requirements, including specific, i.e. T-cell receptor (TCR)-mediated and nonspecific signals. The antigen, recognized via the TCR, has to be presented in the form of peptides bound to the antigen-binding groove of the class II major histocompatability complex (MHC) on the surface of an antigen presenting cell, which delivers accessory signals to the T-cell. Triggering of the TCR/CD3 complex via an appropriate class II-molecule antigenic peptide combination is coupled to phosphoinositol hydrolysis and $Ca^{2+}$ mobilization. A highly cooperative interaction between various nuclear factors, each of which follows a different activation schedule, has to occur to allow activity of the enhancer of the IL-2 gene, thus leading to IL-2 production. More than five sequence motifs to which functionally relevant nuclear proteins bind have been identified in the 5′ flanking region of the IL-2 gene. Analysis of deletion mutants suggests that all the protein-binding sites must be occupied to allow activity of the IL-2 enhancer. Signals that activate only one of the enhancer elements will not give rise to IL-2 transcription.[8–10]

### The Interleukin-2 Receptor

To exert its biologic effects, IL-2 must interact with a specific membrane IL-2 receptor. In contrast to other hormone-mediated systems, cellular activation is a prerequisite for the induction of both the ligand (IL-2) and its receptor (IL-2R), the latter being expressed rapidly at the cell surface in a time-dependent and heterogeneous manner. High-, intermediate- and low-affinity forms of the IL-2R exist, with different dissociation constants.[11–14]

The high-affinity IL-2R is composed of at least two non-covalently linked chains, IL-2Rα and IL-2Rβ, with molecular weights of 55 and 75 kDa, respectively. Analysis of the nucleotide sequences encoding IL-2Rα and IL-2Rβ has shown that the two genes are unrelated. The α-chain of IL-2R consists of an extracellular domain of 219 amino acids, a transmembrane region of 19 amino acids and a short cytoplasmic domain of 13 amino acids. IL-2Rα was formerly called Tac antigen from 'T-activated', since the monoclonal antibody (mAb) that recognizes this molecule was initially determined to recognize an activation antigen on T-cells. Actually, the IL-2R α-chain is termed CD25 antigen according to the cluster of differentiation (CD) nomenclature of surface molecules of haemopoietic cells. In humans, the IL-2R α-chain is encoded by a single gene on chromosome 10 and is induced and expressed only after T-cell, B-cell or monocyte activation. This specific feature of CD25 provides investigators with a unique marker of immune system activation before the appearance of other cell surface determinants and well before lymphocyte proliferation. Mitogen stimulation *in vitro* induces a peak level of approximately 60 000 site/cell within 48–72 h but the number of receptors then progressively declines.[11–21]

The IL-2R β-chain, whose gene is located on chromosome 22, consists of a 214 amino acid extracellular domain, a 25 amino acid transmembrane region and a 286 amino acid cytoplasmic tail. The longer cytoplasmic domain of the β-chain suggests that this chain is involved in efficient signal transduction. Homology comparisons have revealed that the IL-2R β polypeptide corresponds to a member of a novel cytokine receptor superfamily, the haemopoietin receptor family or type-1 cytokine receptor family, which includes the membrane receptors for IL-3, IL-4, IL-6, IL-7, erythropoietin, granulocyte-macrophage colony stimulating factor (GM-CSF), prolactin and growth hormone.[11–14,22,23]

The intermediate affinity receptor contains only the 75 kD IL-2R β-chain, while the low affinity receptor is comprised of the 55 kD IL-2R α-chain. Without the presence of the α-chain, to achieve cell activation IL-2 must be present at concentrations ten- to 100-fold above those required for a physiological response. The rate of association of IL-2 with, and dissociation from, the α-chain is very rapid, whereas the association rate with the β-chain alone is slower and the dissociation rate very slow. Therefore, expression of the high affinity receptor endows the cell with a receptor provided with a fast on rate and a slow off rate, thus ensuring that IL-2 is avidly bound, retained and internalized at physiological concentrations. However, the β-chain of IL-2R is also able to mediate the internalization of bound IL-2 in the absence of the α-chain. In fact, it has been shown that the IL-2R β-chain mediates the initial proliferative response of resting T-lymphocytes and large granular lymphocytes and the initial phase of induction of lymphokine activated killer (LAK) and NK activities. Furthermore, the binding of IL-2 to the p75 $\beta$-chain induces the expression of the IL-2R$\alpha$ gene and the CD25 antigen in these cells. Thus, the inducible nature of IL-2R$\alpha$ gene expression contributes to a transient display of a high-affinity receptor, while the IL-2R$\beta$ protein appears to play a major role in growth signal transduction, although this ability probably requires the coexpression of a novel IL-2R component termed the $\tau$-chain.[11–14,24–28]

In fact, a third subunit of human IL-2R has recently been identified. It is a p64 molecule named the $\tau$-chain, which coprecipitates with the $\beta$-chain and IL-2 in immunoaffinity columns conjugated with TU11, an mAb specific for the IL-2R $\beta$-chain. The IL-2R $\tau$-chain, whose gene has been cloned, belongs itself to the type-1 cytokine receptor family, participates in the formation of the high- and intermediate-affinity IL-2R and it has been shown to be essential for the receptor-mediated internalization of IL-2. In fact, analysis of the IL-2R chains cDNAs in sublines derived from murine fibroblastoid cell lines, showed that coexpression of $\alpha$-, $\beta$-, $\tau$-chains and $\beta$-, $\tau$-chains is required in order to obtain high- and intermediate-affinity binding, respectively, of IL-2 by transfected cells. Moreover, transfected cells expressing $\alpha$-, $\beta$- and $\tau$-chains have been shown to internalize IL-2 more efficiently than cells bearing only $\beta$- and $\tau$-chains, while cells expressing only $\alpha$- and $\beta$-chains have rarely been shown to be able to internalize IL-2 although they have the ability to bind IL-2 with greater affinity than intermediate-affinity receptor bearing cells. In addition, with the use of various methods including coimmunoprecipitation analysis, radiolabelled IL-2 cross-linking and flow cytometric resonance energy transfer measurement, additional proteins such as class-I MHC and intercellular adhesion molecules ICAM-1 were found to be associated with IL-2R. Furthermore, the intracellular domain of IL-2R$\beta$ is associated with a tyrosine kinase, lck, which, after IL-2R activation, phosphorylates several specific proteins involved in cell proliferation[14,23,29,30] (Table 1).

The development of anti-Tac and anti-Mik-$\beta$1 mAb to the $\alpha$- and $\beta$-subunits of IL-2R, respectively, together with Northern blot analysis using chain-specific probes, has resulted in the delineation of IL-2R expression on different cell types. By using these mAbs, a marked difference in expression of IL-2R subunits on blood CD4+ and CD8+ T-cells has been demonstrated between adults and newborns. In the adult blood, reciprocal expression of IL-2R$\alpha$ and IL-2R$\beta$ has been observed in CD4+ and in CD8+ T-cells. Some CD4+ T-cells expressing IL-2R$\alpha$ have been detected, but IL-2R$\beta$, CD4+ cells were few. On the other hand, CD8+ T-cells express significant IL-2R$\beta$, but little IL-2R$\alpha$. Although the reason is still unknown, it is probably because the differences in the IL-2R subunits expression are related to the biological role of T-cell subsets. Both CD4+ and CD8+ T-cells from the newborns, which probably consist mainly of naive populations, showed only negligible expression of IL-2R subunits. IL-2R subunits have appeared to be preferentially expressed on CD4+ and CD8+ T-cells with memory phenotypes in the adult blood. Isolated memory (CD45RO+) CD4+ and CD8+ T-cells, unlike naive (CD45RO−) ones, were able to proliferate in response to exogenous IL-2 as well as to the recall antigen. This suggests that IL-2R subunits expressed on circulating T-cell subsets might play an important role in eliciting a secondary response. According to the significance of IL-2 for T-cell growth, it is plausible to suppose that memory T-cells could readily respond to recall antigens, their already expressed IL-2R subunits being largely involved in antigen-induced proliferation.[31]

**Table 1.** Human IL-2R

| | Chain IL-2R$\alpha$ | IL-2R$\beta$ | IL-2R$\tau$ |
|---|---|---|---|
| Molecular weight (kDa) | 55 | 75 | 40 |
| Amino acid residues ('mature' proteins) | 251 | 525 | 347 |
| Chromosome | 10p 14→15 | 22q 11.2→12 | ND |
| IL-2R (low affinity) | + | | |
| IL-2R (intermediate affinity) | | + | + |
| IL-2R (high affinity) | + | + | + |

Approximate molecular weight of mature proteins (kDa).
The high-affinity receptor is unusual among the cytokine receptors because it consists of three distinct subunits, instead of two subunits.
For references see text.

Finally, the ratio of low-affinity sites to high-affinity sites is approximately 10:1 in both resting and activated lymphocytes and in most IL-2R expressing T-cell lines tested. The majority of NK cells (CD16+) constitutively express the intermediate-affinity IL-2R instead and a subpopulation of NK (CD16−) cells expresses the high-affinity receptor. Human monocytes that respond to IL-2 with induction of IL-1 mRNA and an enhanced cytotoxic capacity, express the $\beta$-chain of IL-2R, but not the $\alpha$-chain (see below).[28,32–36]

IL-2R expression is not limited to normal mononuclear cell populations. The presence of IL-2R has been demonstrated on the membrane of malignant cells in Hodgkin's disease and B- and T-cell neoplasias. The biologic function of IL-2R$\alpha$

on malignant cells is largely unknown, but it is thought to involve the stimulation of cell proliferation.[37–41]

As stated for the IL-2 gene, for IL-2Rα an interaction between various nuclear factors also has to occur to allow the activity of the gene enhancer. The promoter region of the IL-2Rα gene consists of a minimum of five positive regulatory elements and at least one negative element. IL-2 and IL-2Rα share at least one regulatory element. This datum may explain why these two gene products are usually coexpressed. Unlike the IL-2 gene, which is strictly dependent on triggers from the antigen receptor plus accessory signals, IL-2Rα is expressed after activation with IL-1, IL-5, phorbol myristate acetate or binding of ligands to the TCR alone, without a requirement for second signals.[10]

### Soluble Interleukin-2 Receptor

In 1985 it was first observed that, after *in vitro* cellular activation, not only the expected cell-associated IL-2R, but also a soluble form of the receptor could be found in the cell-free supernatants of these cultures. This soluble molecule seems to correspond to a truncated extracellular part of the membrane bound Tac antigen, is smaller than its cellular counterpart (45 vs 55 kDa) and retains the ability to bind IL-2. Similarly to cellular IL-2R expression, soluble IL-2R (sIL-2R) production requires *de novo* synthesis, but not cellular proliferation.[42]

To study sIL-2R, the anti-TAC mAb and an mAb termed 7G7/B6, which binds to the human IL-2R at an epitope distinct from that recognized by the anti-Tac, have been used to construct a 'sandwich' enzyme-linked immunoasorbent assay (ELISA) that offers a simple and rapid method for quantitating sIL-2R levels. Although both mAbs recognize the IL-2R, anti-Tac, but not 7G7/B6, blocks IL-2 binding and the binding of 7G7/B6 to the IL-2R α-chain is not blocked by anti-Tac or IL-2.[21,43]

Enzymatic digestion of this molecule reveals that it is a complex glycoprotein, containing both N- and O-linked sugars and sialic acid residues, similar to those previously demonstrated on the mature cell surface IL-2R. The generation of soluble IL-2R does not appear to be the result of cell death with subsequent IL-2R release, since IL-2R-positive cells killed by repetitive freezing and thawing and placed back into culture do not release sIL-2R.[42,44]

Theoretically, several mechanisms might account for the production of sIL-2R. One mechanism refers to the possibility that separate genes encode the secreted and cellular forms of IL-2R. Alternatively, both forms of the IL-2R could be the product of the same gene via differential mRNA splicing, giving rise to an 'anchor minus' protein which is then released. However, the predominant mechanism of release appears to be, at least in T- and B-cells, the proteolytic cleavage at the cell surface. Since the sIL-2R has been detected in the supernatants of activated cells cultured in serum-free media, a cellular rather than an exogenous protease should be responsible for the release of the soluble form of the receptor. However, the rate of release of this molecule is in proportion to its cell surface expression and any cell expressing the α-chain protein seems to be capable of releasing sIL-2R. In addition, sIL-2R has not been found in the complete absence of concurrent or temporally related cell-surface Tac expression, although, *in vivo*, cells expressing the membrane-associated molecule might not necessarily be detected within the same physical compartment.[42–47]

Accordingly, in a recent study it was observed that following mitogen stimulation *in vitro*, 25% of CD4+ lymphocytes and 19% of CD8+ lymphocytes expressed CD25. Approximately 55% of CD25 positive MNCs were CD4+ lymphocytes and 45% were CD8+ lymphocytes. A significant correlation was demonstrated between the membrane bound IL-2R and its soluble form in supernatants.[48]

The generation of sIL-2R is uniformly 'activation-dependent' except in specific neoplastic conditions in which the malignant phenotype is characterized by the constitutive expression of Tac and release of sIL-2R. Activated normal peripheral blood MNCs (T-cells, B-cells and monocytes) as well as certain T- and B-cell lines have all been found to release the soluble form of the IL-2R.[35,36,41,42,46,49]

*In vitro* stimulation with lipopolysaccharide initiates monocytes to produce the IL-2R light chain. After 48 h, considerable quantities of sIL-2R are produced by activated monocytes. Since the main portion of the receptor is present in the cytoplasm, it is probable that in monocytes cell-associated p55 IL-2R is not necessarily attached to membranes, but is present in a soluble form in the cytoplasm, presumably freshly produced with the aim of being secreted. Incidentally, this explains why induction of p55 IL-2R does not lead to high-affinity binding by monocytes as observed with T-cells: the IL-2R α-subunit is hardly expressed on the plasma membrane.[36]

The generation of sIL-2R is more rapid and efficient in cultures stimulated with the polyclonal activators, compared with those stimulated with either soluble exogenous antigens or cell-associated alloantigens. This difference is presumably related to the precursor frequency of the cells being activated and is in accord with previous determinations of cell surface IL-2R expression after activation with various stimuli.[42,44]

In supernatants of cultured cells, sIL-2R has also been shown to be present in normal sera. The normal levels of serum sIL-2R (expressed as arbitrary units referred to a standard preparation of supernatants from phytohaemoagglutinin (PHA)-stimulated lymphocytes; 3 units = 1 pg of purified protein) have been shown to range between 100 and 500 U/ml, the mean value being approximately 250 U/ml. These values are likely to reflect the ongoing lymphocyte activation which normally occurs upon physiological stimuli. Age-related changes in serum sIL-2R levels in otherwise healthy subjects have been described. Initial studies disclosed that levels of sIL-2R in cord blood and peripheral blood from normal adults were comparable. It is likely that cord blood sIL-2R might be the result of lymphopoiesis or be of maternal origin rather than of exogenous antigenic stimulation. Subsequently, it has been found that serum sIL-2R levels are significantly higher in healthy children younger than 6 years old than in normal adults, gradually declining to typical adult levels by 10 years of age and then rising again in elderly persons, suggesting a higher baseline level of immune activation during childhood and ageing. No significant sex-related differences have been noted. Recently, one study performed on 228 healthy young adult blood donors has confirmed that there is no correlation between serum levels and the different ages or sex of adult donors. Another study performed on a small number of subjects has instead demonstrated that sIL-2R displays a pronounced circadian phase-dependency.[47,49–55]

An immunoreactive soluble form of IL-2R has also been found in the urine of normal individuals. Like serum sIL-2R, the urinary receptor has a molecular weight of 40–45 kDa and specifically binds IL-2. Moreover, the urine levels of sIL-2R correlate positively with those in the serum. Currently available literature does not point to any definitive conclusion about the renal handling of sIL-2R. However, the comparative data with the $\beta$-2 microglobulin may be consistent with the hypothesis that sIL-2R undergoes glomerular filtration and partial tubular reabsorption as well as the $\beta$-2 microglobulin. On the other hand, it could also be that sIL-2R is produced, at least in part, within the urinary tract. Indeed the finding that glomerular mesangial cells are able to secrete IL-1 is in agreement with this hypothesis, since IL-1 is involved in inducing the expression of the CD25 molecule on the lymphocyte surface.[10,56–58]

Increased levels of the soluble form of IL-2R$\alpha$ have been observed in the serum of patients with malignant, autoimmune and allergic disorders, as well as in subjects affected by systemic infectious diseases or undergoing allograft rejection (Table 2). Although *in vitro* the $\alpha$-chain of IL-2R is released from the cell membrane of activated B-cells and monocytes by proteolytic cleavage, the amount released has been thought to be trivial compared to T-lymphocyte production. Thus, the presence of the soluble form of the IL-2R $\alpha$-chain in serum has been suggested to mostly reflect the state of T-cell activation in these subjects. Depending on the type of disorder and on clinical state, increased levels of sIL-2R can be detected in other body fluids as well.[46,49,55,59–61]

**Table 2.** Diseases or states with highserum sIL-2R levels

| |
|---|
| Hairy cell leukaemia |
| Adult T-cell leukaemia |
| B-cell chronic lymphocytic leukaemia |
| Non-Hodgkin's lymphoma |
| Hodgkin's disease |
| Acute lymphoblastic leukaemia |
| Myelodysplastic syndromes |
| Multiple myeloma |
| Acute myeloid leukaemia |
| Cutaneous T-cell lymphomas |
| Angioimmunoblastic lymphadenopathy |
| Lung, colon, stomach or uterine cancer |
| Nasopharyngeal carcinoma |
| Systemic lupus erythematosus |
| Rheumatoid arthritis |
| Juvenile rheumatoid arthritis |
| Polymyositis |
| Sjorgren's syndrome |
| Myastenia gravis |
| Insulin dependent diabetes mellitus |
| Graves' disease |
| Toxic multinodular goitre |
| Multiple sclerosis |
| Crohn's disease |
| Ulcerative colitis |
| Coeliac disease |
| Sarcoidosis |
| IgA nephropathy |
| Atopic dermatitis |
| Psoriasis |
| Anaphylactic reaction to food |
| Systemic sclerosis |
| Kawasaki disease |
| Infectious mononucleosis |
| Measles |
| Virus hepatitis |
| AIDS |
| Plasmodium falciparum infection |
| Chronic hepatosplenic schistisomiasis |
| Strongyloidosis |
| Fasciolosis |
| Alveolar echinococcosis of the liver |
| Visceral leishmaniasis |
| Leprosy |
| Tubercolosis |
| Burn |
| Common variable immunodeficiency |
| Haemodialysis |
| Transplantation |

For references see text.

Although investigations of IL-2-mediated growth effects have been focused on the interaction of the cytokine at the cell surface receptor, it is possible that the released receptor plays an immunomodulating role. Although the released form of sIL-2R is 10 kDa smaller due to lack of transmembrane and intracytoplasmic domains, it retains the ability to bind IL-2. In fact by affinity chromatography, sIL-2R is capable of binding to purified recombinant IL-2 (rIL-2). It has a similar affinity to the Tac surface protein, which, as previously stated, is approximately 100- to 1 000-fold lower than the high-affinity IL-2R complex. Experiments examining the addition of the purified natural or synthetically generated sIL-2R protein to *in vitro* IL-2 functional assays show inhibition of the stimulatory effects of exogeneously added IL-2 (see below).[44,49,55,60–62]

### Increased sIL-2R Levels in Haemopoietic Malignancies

Increased serum sIL-2R levels have been found in a variety of lymphoproliferative and haematologic malignancies, including hairy cell leukaemia, adult T-cell leukaemia, B-cell chronic lymphocytic leukaemia, acute leukaemia, non-Hodgkin's lymphoma, Hodgkin's disease and myelodysplastic syndromes. Although the increase in serum levels may result from release by activated normal cells, the levels are more likely to derive, in most cases, from neoplastic cells. In fact, as discussed by Rubin and Nelson,[49] in haemopoietic malignancies, increased sIL-2R levels are mostly indicative of a malignant phenotype associated with deregulated or enhanced expression of CD25. Nevertheless, in the interpretation of these measurements, one should consider that host cellular immune response may contribute to the generation of sIL-2R. However, they generally correlate with disease progression and/or with response to therapy, so that the measurement of serum sIL-2R may be a useful index of activity and extent of the disease.[49,55,59–61]

The increased serum sIL-2R levels are not specific of any haematological disorder, nevertheless very high levels of sIL-2R (up to 50 000 U/ml) may be considered diagnostic of hairy cell leukaemia (HCL), in the context of the clinicopathological picture of the disease.[63–66] Similar levels may be detectable in adult T-cell leukaemia (ATL), but in the presence of peculiar epidemiological and clinical features.[41,56,67–71] Human T-lymphotropic virus type-1-associated (HTLV-1) ATL is a malignancy of CD4+ T-cells. The constitutive expression of high levels of CD25 on malignant transformed cells has proved to be an extremely valuable phenotypic marker distinguishing ATL from other histologically similar lymphoreticular neoplasms, such as Sezary syndrome and overexpression of the IL-2R α-chain is considered to be one of the characteristics of T-cells transformed by HTLV-1. In fact, essentially all HTLV-1 infected T-cell lines established from ATL patients or HTLV-1 carriers, as well as the majority of the ATL leukaemic cells, constitutively express IL-2Rα mRNA and protein IL-2Rα overexpression is tightly associated with T-cell immortalization and/or activation of HTLV-1 infected T-cells. Accordingly, very high serum sIL-2R levels have been found in this disease. Acute patients have the highest serum levels, whereas those with smouldering disease or healthy HTLV-1-antibody-positive carriers have normal to slightly elevated levels. Intermediately elevated levels are seen in patients with the chronic form of the disease. Moreover, serum sIL-2R levels reflect disease activity. In fact, these values decrease to normal when patients respond to treatment and increase in progressive disease. Serum sIL-2R levels appear to reflect the total tumour burden accurately, irrespective of circulating leukaemic cell numbers, which are sometimes low even in the presence of significant disease. Serial measurements of sIL-2R levels thus seem to be a useful, non-invasive laboratory method in the management of these patients.

As stated above, high serum sIL-2R levels have also been detected in patients with HCL,[63–66] a chronic lymphoproliferative disease of B-cell origin. Release of the receptors by hairy cells appears to be the most likely explanation for this finding. Serum values fell to near the normal range in patients responding to therapy, whereas no significant changes have been found in patients who failed to respond. Hence, the determination of sIL-2R levels may be important in monitoring disease activity and response to treatment.

Most adults with B-cell chronic lymphocytic leukaemia (B-CCL)[72–74] have increased serum sIL-2R levels, the higher being observed in patients with more advanced disease. Purified malignant B-cells from such patients release sIL-2R after *in vitro* culture, although in some cases the membrane receptor is not expressed on malignant cells. By contrast, little or no detectable sIL-2R is released by purified T-cells from these patients. The sIL-2R in patient serum is derived largely from the 'activated' malignant cells rather than from the normal activated T-cells.

In children with newly diagnosed acute lymphoblastic leukaemia,[74–79] increased values of sIL-2R have been found, the levels being lower in T-cell leukaemias, than in non-T, non-B cases. Among the patients with non-T-non-B leukaemia, higher serum receptor levels have been shown to be associated with a poorer treatment outcome. Further, serum sIL-2R levels contributed independent prognostic information. The source of sIL-2R in patients with acute lymphoblastic leukaemia is unknown.

It has been observed that in patients with non Hodgkin's lymphoma (NHL),[51,80–82] high serum sIL-2R levels correlate with more advanced disease and with a poorer clinical outcome. Serum sIL-2R levels have been shown to be elevated in all of the cases of T-cell and most of the cases of B-cell lymphoma examined. Higher serum sIL-2R levels are also related to an increased likelihood of treatment failure. More importantly, the serum sIL-2R level seems to be an independent prognostic factor. The measurement of serum sIL-2R in children with NHL should thus improve existing methods of risk assignment. That serum sIL-2R in this disease is largely derived from tumour cells is suggested by the correlation of sIL-2R levels with tumour burden, the high levels of receptors found in malignant serous effusions and the fact that some lymphoma cell lines have high levels of cell-associated IL-2R.

In adults with Hodgkin's disease (HD),[59–61,83–85] it has been found that higher serum sIL-2R levels are associated with more advanced stages of disease and with the presence of constitutional symptoms. Serum sIL-2R levels returned to normal in patients who responded to treatment, but persisted elevated or increased in patients with resistant or progressive disease. In addition, the sIL-2R level may independently predict treatment outcome. Because the majority of Reed–Sternberg cells and their mononuclear variants strongly express IL-2R, these high serum levels stem at least in part from release of the receptor by malignant cells. Therefore, serum sIL-2R levels reflect tumour burden. However, unstimulated MNCs of patients release more sIL-2R than controls, suggesting that the host cellular response contributes to sIL-2R production. No difference was observed between PHA-stimulated MNCs of patients and controls.

Significant increase of sIL-2R in a group of patients with myelodysplastic syndromes[86] has been described. Interestingly, six patients who had been under treatment with recombinant GM-CSF for at least 2 weeks, demonstrated a three- to seven-fold increase of serum sIL-2R compared to pretreatment levels. These data, however, cannot suggest whether increased sIL-2R release is a primary event due to involvement of lymphocytes in the malignant clone or whether it results from secondary alteration of the cytokine network.

Increased serum sIL-2R levels have also been observed in patients with multiple myeloma[87] (the sIL-2R concentration was significantly correlated with the concentration of monoclonal immunoglobulin in serum), acute myeloid leukaemia[59,60] (higher levels were observed in cases with M4–M5 morphology as compared to patients with M1–M2–M3), angioimmunoblastic lymphadenopathy[59] and cutaneous T-cell lymphomas (CTCL).[88–90] Although stable chronic myelogenous leukaemia[91,92] is not associated with increased serum sIL-2R, during the blastic phase of chronic myelogenous leukaemia, CD25 antigen is expressed on malignant cells and elevated sIL-2R levels can be detected in serum. Rising serum sIL-2R levels antedate clinically apparent blast crisis in this condition.

The biological role of sIL-2R in haematological malignancies needs further evaluation. However, theoretically sIL-2R can affect a number of IL-2-dependent functions and, thus, it might indeed be responsible for several alterations of immune functions commonly found in haematological malignancies.

A finding supportive of this possibility is the observation that an inverse relationship of serum sIL-2R concentrations to the *in vitro* NK activity in patients with HCL exists. Furthermore, lymphocytes of B-CCL patients with the lowest serum sIL-2R levels show the best mitogenic response and helper capacity.[65,74]

Finally, in patients with cutaneous T-cell lymphoma, the decrease of NK activity correlates with the augmentation of serum sIL-2R. After a 4 day stimulation with IL-2, MNCs from CTCL-affected patients show an increase of cytotoxic activity similar to that of healthy donor MNCs. Normal donor MNCs demonstrate a diminished IL-2-induced cytotoxic activity in 25% of CTCL sera compared to control sera, while IL-2-dependent proliferation of 48 h PHA blasts is lower in CTCL sera than in control sera. Enrichment of media with exogeneous sIL-2R inhibits the IL-2-dependent generation of cytotoxic activity and mitogen blast proliferation suggesting that elevated sIL-2R levels account for diminished NK activity by neutralizing IL-2 in CTCL patients.[90]

### sIL-2R Release in Other Malignancies

High blood levels of sIL-2R may be found in patients with solid neoplasms. Moreover, it has been suggested that sIL-2R levels in the blood of patients with cancer may display a prognostic significance, probably related to host immune reactions rather than representing a tumour marker.[93]

High serum sIL-2R levels have been found in patients with lung cancer[94–96] of different histological type (small cell, epidermoid carcinoma, adenocarcinoma, unclassified carcinoma). No significant differences were found within different histological types, nor within different disease stages. However, it seems that sIL-2R changes after surgery have prognostic importance, compared to the levels found before surgery. In fact in patients affected by epidermoid carcinoma or adenocarcinoma, a surgery-induced increase in sIL-2R levels was seen 7 days after surgery in most of the patients studied. On the thirtieth day after surgery, sIL-2R values were lower than the preoperative values in more than half patients and greater in the remaining ones. After a median follow-up of 10 months, the latter group showed a significantly higher relapse rate, thus suggesting that the persistence of increased sIL-2R levels in the postoperative period is associated with a higher early relapse rate in patients with operable non-small cell lung cancer.

In patients with nasopharyngeal carcinoma[97] serum sIL-2R levels have been shown to be elevated and to correlate with clinical staging. Higher sIL-2R levels have been observed in patients with bone metastasis, but not in patients with intracranial involvement. Since depressed cell-mediated immunity is well-documented in patients with nasopharyngeal carcinoma, it has been suggested that sIL-2R may serve as a blocking factor that competes with IL-2 function, resulting in a decreased mitogenic response.

Soluble IL-2R levels assessed in sera derived from patients with non-metastatic or metastatic breast cancer[98] or from healthy controls did not differ significantly from each other. When sIL-2R levels were assessed in supernatants from mitogen-stimulated MNCs derived from either patients or healthy controls, healthy individuals were found to produce sIL-2R in an amount that was significantly higher than levels found in both patient groups, i.e. with non-metastatic as well as with metastatic disease under immediate cytostatic treatment. Moreover, a significant difference was found in mitogen-stimulated sIL-2R production between patients with or without metastases, the first group being more depressed. Adjuvant radio- and chemotherapy both resulted in a significant and long-lasting depressive effect upon mitogen-induced sIL-2R release. A strong correlation was found between mitogen-induced sIL-2R concentrations and results obtained in simultaneous experiments assessing mitogenic stimulation of MNCs.

Serum levels of sIL-2R have also been shown to be significantly increased in patients affected by cancer of the colon, stomach or uterine cervix and metastatic cancer patients showed significantly higher values than the non-metastatic ones. Thus, serum sIL-2R levels in non-haemopoietic malignancies may also be increased, probably as a result of activation of the immune system in response to cancer. Moreover, there is some evidence that serum sIL-2R levels may be an indicator of metastasis for patients with solid tumours.[93,99]

Finally, in patients with progressive metastatic renal carcinoma, malignant carcinoma, colorectal cancer, B-cell lymphoma or HD treated with rIL-2, significant increases in sIL-2R levels have been observed when comparing values on day zero and after treatment course. Interestingly, sIL-2R correlated with CD25 positive blood MNCs.[100,101]

### sIL-2R Production in Autoimmune Diseases

Recent papers have demonstrated increased levels of sIL-2R in the sera of patients with a variety of autoimmune or immune-mediated diseases including systemic lupus erythematosus, rheumatoid arthritis, juvenile rheumatoid arthritis, polymyositis, Sjogren's syndrome, myastenia gravis, insulin-dependent diabetes mellitus, Graves' disease, multiple sclerosis, Crohn's disease, ulcerative colitis, coeliac disease, sarcoidosis, IgA nephropathy, atopic dermatitis and psoriasis. In most diseases serum levels correlate with disease activity as defined by various clinical and laboratory parameters. These data suggest the usefulness of measuring serum sIL-2R levels in the management of autoimmune patients.[102]

However, in systemic lupus erythematosus (SLE),[103–108] it has not been possible to find any association between serum sIL-2R and a particular clinical manifestation. Furthermore, serum sIL-2R levels have very frequently been found to be increased in active as well as inactive SLE. In fact, the concentration of sIL-2R was higher in inactive SLE patients than in normal controls and was significantly increased in active as compared to inactive SLE patients. When patients with active disease were followed up serially, it was found that the sIL-2R concentration fell when the disease became inactive. However, there was no statistically significant association between sIL-2R and the grades of disease activity, neither did sIL-2R levels parallel indicators of serological disease activity such as anti-DNA antibodies. On the whole these data suggest that lymphocyte activation may still be present even though the disease is considered inactive under clinical criteria and that sIL-2R and autoantibodies measure different facets of immune system activation.

Serum levels in rheumatoid arthritis (RA)[109–116] patients are significantly raised compared to patients with non-inflammatory joint disease and to age-matched, disease-free controls. Moreover, within the RA patients, synovial fluid sIL-2R levels are significantly higher than serum levels, indicating that proliferating synovial tissue is the probable source of the sIL-2R detected in the circulation. In sequential studies of individual patients during spontaneous remission, the serum sIL-2R levels correlate with disease activity. Serum concentrations of sIL-2R are correlated with erythrocyte sedimentation rates and C-reactive protein levels. Interestingly, the reduction of sIL-2R levels towards control values precedes clinical remission, suggesting that this is not a secondary event reflecting clinical improvement, but is more likely to be related to the activation of immunopathogenic mechanisms that produce inflammation. Consistent with this hypothesis is the high spontaneous production of sIL-2R in freshly isolated MNCs from RA synovial exudate. Similar levels can only be achieved by autologous blood MNC populations after stimulation with mitogens. Of great interest is the highly significant correlation observed between synovial fluid levels of sIL-2R and IL-1$\beta$, since IL-1 has been implicated as a pathogenic mediator in articular diseases. The correlation noted could then result from the action of IL-1 as a cofactor in CD25 expression. Moreover, since synovial fluid sIL-2R levels are significantly elevated when compared with those of other osteoarthritic conditions, this suggests that their determination may be useful in a clinical situation where a definitive diagnosis has not yet been possible. In contrast, PHA-stimulated MNCs derived from patients with RA produced similar amounts of sIL-2R as compared to PHA-stimulated MNCs derived from healthy controls. No significant correlation of sIL-2R levels in sera with sIL-2R concentrations in supernatants of mitogen-stimulated MNC has been found. Spontaneous, i.e. non-mitogen-stimulated, production of sIL-2R by MNCs in culture was below the detection limit in patients with RA as well as in controls. Furthermore, the relationship between synovial fluid MNC production of sCD8, sCD4 and sIL-2R has been examined. The results reveal two populations of patients. Synovial fluid MNCs from one population produced high levels of sCD8 and relatively low levels of sIL-2R, whereas the other population produced low levels of sCD8 and high levels of sIL-2R. Taken together, the data indicate that the size or activity of the CD8+ T-cell populations in the rheumatoid synovium is inversely related to the activity of CD25 positive MNCs. The observed results could be the expression of fluctuations in the activity of the two different populations of MNCs, one of which, largely CD8+, mediates remission, while a population expressing high levels of CD25 mediates an inflammatory response resulting in an increase in disease activity. Synovial fluid levels of sCD4 correlate positively with sIL-2R levels, but no correlation has been found with sCD8 levels. The levels of serum sCD4 in these patients closely follow the sIL-2R levels, possibly indicating that they may be derived from the same cellular subsets. In fact, studies of synovial fluid MNCs show that the release of these two molecules occur at a similar rate. However, the serum sIL-2R level in RA probably reflects activation of underlying immunopathogenic mechanisms and appears to be an excellent monitor of clinical disease activity. More importantly rising levels may also predict exacerbation of disease activity.

In juvenile rheumatoid arthritis (JRA)[117] increased sIL-2R levels have also been observed. The highest values have been seen in patients with systemic JRA and in patients with clinically active disease, but serum sIL-2R levels were elevated in all subgroups of clinically active patients compared to controls.

In polymyositis[118,119] the combination of elevated sIL-2R and sCD8 levels has been associated with active muscle inflammation. Serial measurements show a rise in serum sIL-2R and sCD8 levels before clinical and biochemical relapse.

In Sjogren's syndrome[102] the serum levels of sIL-2R progressively increase from patients with disease confined to the exocrine glands to patients with extraglandular manifestations without pseudolymphoma or lymphoma and, finally, to patients with lymphoproliferative diseases. Therefore, high serum sIL-2R levels seem to indicate the progression of disease to extraglandular involvement and to pseudolyphoma or lymphoma. Moreover, further studies are needed to obtain a clear-cut demonstration that monitoring of serum sIL-2R provides a useful predictive index for the development of lymphomas which frequently complicate the disease course.

In myastenia gravis[120] the sIL-2R titre is high in a significant number of patients, especially when tested before thymectomy. Patients with a severe form of the disease present the highest sIL-2R serum levels and a significant and progressive decline of sIL-2R titres is observed after thymectomy, which is well-correlated to clinical improvement in individual patients. These findings, taken together, suggest that the evaluation of sIL-2R in the serum may represent a good marker of disease severity and of the effect of thymectomy in the follow-up of individual patients.

In insulin dependent diabetes mellitus (IDDM)[121,122] newly diagnosed patients' sera, higher levels of sIL-2R in comparison with sera from healthy subjects have been observed. After a time interval from the beginning of symptoms of type 1 diabetes and exactly 6 months after clinical diagnosis of the disease, the patients maintained levels which were high with respect to healthy subjects and were almost identical to the ones at onset. In contrast mitogen stimulated MNCs from both newly diagnosed and long-term IDDM patients produce low amounts of sIL-2R. The combined data clearly establish that MNCs from newly diagnosed IDDM patients *in vivo* are activated and have the capacity to express and release increased amounts of sIL-2R, but *in vitro*, with appropriate stimulation, they have decreased capacity to produce such receptors and have defective IL-2 production. The phenomenon of decreased production of IL-2 and sIL-2R *in vitro* is also partially present in long-term IDDM patients and may be of great interest. In this connection, the theory of a generic exhaustion phenomenon (see concluding remarks) cannot be applied to long-term type 1 diabetic patients. When all of these data are considered, the IL-2 and sIL-2R defects in type 1 diabetes seem to be linked to the immunogenetic profile of the disease.

Serum sIL-2R levels have been observed to be significantly increased in newly diagnosed Graves's disease[123–126] patients as compared with controls. The sIL-2R levels were higher in patients with active infiltrative ophthalmopathy than in those without eye symptoms. In patients treated with methimazole for at least 12 months, sIL-2R levels were normalized in the majority of patients without ophthalmopathy, but not in those with ophthalmopathy. Furthermore, a correlation has been found between sIL-2R levels and anti-TSH receptor antibodies, but not with other immune parameters examined. However, it has been suggested that the level of sIL-2R is not only dependent on immunological conditions, but also on thyroid hormone status. In fact, when thyroid hormone is administered to subjects in remission from Graves' disease and in normal controls, the sIL-2R levels significantly increase in both groups. Moreover, the mean level of sIL-2R in patients with toxic multinodular goitre[125,126] is also significantly higher than in normal controls. Thus, sIL-2R levels are higher in the sera of subjects with elevated levels of thyroid hormone, irrespective of the cause of hyperthyroidism. These high serum sIL-2R concentrations might merely be the result of an accelerated turnover of T-cell membranes due to a hypermetabolic state. This phenomenon prevents the use of sIL-2R as a reliable marker of autoimmune activation in hyperthyroid conditions.

Elevated serum sIL-2R levels have been reported in patients with multiple sclerosis (MS)[127–130] of the chronic progressive type, during a period of relative disease quiescence. This finding, taken in conjunction with that of elevated serum IL-2 levels and the evidence of elevated and prolonged expression of cell-bound IL-2R, indicates that an activated cellular immune state parallels the progression of the demyelinating process in MS. Soluble IL-2R levels in both serum and cerebrospinal fluid have been found to be higher in patients with relapsing MS than in patients with disease quiescence. Furthermore, sIL-2R levels change with high sensitivity in parallel with disease activity. Levels of sIL-2R in steroid-treated chronic progressive patients were markedly lower than in untreated chronic progressive patients and were comparable to healthy controls.

Levels of serum sIL-2R have been shown to be significantly higher in Crohn's Disease (Cd) and ulcerative colitis (UC).[131–135] Intestinal mucosal MNCs always produced more sIL-2R than peripheral cells. Spontaneous sIL-2R production by mucosal MNCs is significantly elevated in Cd, but not in UC supernatants. A positive correlation has been found between blood sIL-2R and spontaneous production by intestinal MNCs of Cd patients and surgical control patients, whereas UC plasma sIL-2R correlated with spontaneous production by peripheral MNCs. Upon stimulation with mitogens, Cd, normal controls and diverticulitis lamina propria MNCs reached similar maximal sIL-2R secretion levels, while UC lamina propria mononuclear cells secreted significantly less sIL-2R. On the other hand, it has been found that endoscopical mucosal biopsy specimens from patients with inflammatory bowel diseases contained significantly increased amounts of sIL-2R. Furthermore, the highest concentrations were consistently found in the most inflamed biopsy specimens. In Cd, increased blood sIL-2R levels have been detected in patients with more clinically severe disease. A progressive increase in sIL-2R levels has been noted to correlate with endoscopic measurement of disease extent, while sIL-2R levels did not correlate with other markers of systemic lymphocyte activation, suggesting possible local mucosal production. Sequential determinations in individual patients revealed a good correlation between sIL-2R, clinical course and laboratory measurements of disease activity including the C-reactive protein and the erythrocyte sedimentation rate. More importantly, elevated levels of sIL-2LR preceded clinical relapse of asymptomatic patients.

Concentrations of sIL-2R in the serum of patients with coeliac disease[136] are significantly raised in patients with untreated disease compared with treated patients and controls. Longitudinal studies in individual coeliac patients showed that serum sIL-2R fell following commencement of a gluten-free diet. Gluten challenge of treated coeliac patients for 1 week resulted in a significant increase in serum sIL-2R, which returned to prechallenge levels within 4 weeks of recommencement of a gluten-free diet. These data suggest that serum sIL-2R levels in patients with coeliac disease reflect specific immunological activation in response to gluten ingestion. Measurement of serum sIL-2R may therefore be useful in the assessment of response to treatment in patients with coeliac disease.

Elevated levels of sIL-2R have been found in patients with active pulmonary sarcoidosis[137,138] as compared to normal controls. Furthermore, serum sIL-2R levels have been shown to fall after clinical improvement following corticosteroid treatment, suggesting that measurements of serum sIL-2R could prove useful in monitoring disease activity. Moreover, it has been observed that the levels of sIL-2R in the serum of sarcoidosis cases with bilateral hilar lymphoadenopathy (BHL) are significantly higher than those of cases without BHL.

In patients with IgA nephropathy[139] serum levels of sIL-2R were significantly higher than in controls and were higher in the subgroup of patients with episodic macrohaematuria. Since the presence of red blood cells in the urinary sediment has been shown to be closely related to serum sIL-2R levels, measurement of sIL-2R may provide a good marker for disease activity.

Serum sIL-2R levels are also significantly elevated in subjects with atopic dermatitis[140] and psoriasis[140] as compared to healthy controls. Furthermore, sIL-2R levels in atopic dermatitis patients showed a significant correlation with IgE levels and body surface involvement and longitudinal studies have shown that measurement of serum sIL-2R may have a prognostic value. Significant elevation of sIL-2R was also observed in sera from children with histories of anaphylactic reaction to food,[141] as compared to non-allergic controls. Finally, also in systemic sclerosis[142–144] and in Kawasaki disease[145] high levels of serum sIL-2R have been reported.

It is intriguing to note that in two groups of subjects, i.e. healthy old individuals and young HLA-B8,DR3 positive subjects, known to show an increased incidence of autoimmune diseases or phenomena, a decreased *in vitro* production of sIL-2R by PHA-stimulated MNCs has been observed. In fact, after *in vitro* stimulation, cultures from both HLA-B8,DR3 positive individuals and elderly subjects are characterized by hyposecretion of IL-2 and sIL-2R as compared with cultures from HLA-B8,DR3 negative subjects and young individuals, respectively. By contrast, serum sIL-2R levels are increased in these subjects.[146,147]

In summary, the discussed findings suggest that in autoimmune diseases sIL-2R levels may represent a good marker of disease activity which indirectly reads the ongoing activation of immunoreactive cells which are involved in the pathogenetic events of these immunomediated conditions.

With regard to the degree of activation required to increase serum levels of sIL-2R, serum sIL-2R levels have recently been measured in healthy subjects after immunization with keyhole limpet haemocyanin (KLH). Despite induction of strong antibody responses, KLH immunization did not result in consistent elevations of sIL-2R levels. This datum suggests that inflammatory diseases in which elevated sIL-2R levels have been noted, involve more extensive stimulation of immune cells, either in number or in degree.[148]

Although it has been suggested that sIL-2R has a role in down-regulating IL-2 dependent responses, it remains to be defined whether circulating sIL-2R intervenes in inducing the immunologic dysfunctions commonly found in autoimmune diseases. In this regard, it has to be remembered that sIL-2R in synovial fluids has been demonstrated to compete with cell-associated receptors for available IL-2. In fact sIL-2R levels in synovial fluid correlate with functional inhibition of IL-2-driven responses assessed as the inhibition of an *in vitro* response to optimal concentration of rIL-2.[109]

### sIL-2R Release in Infectious Diseases

Viruses, bacteria and parasites may actively engage the immune system by inducing a strong activation, thus it is not surprising that in infectious diseases it is possible to observe an increase of sIL-2R blood levels. Moreover, little clinical use may result from sIL-2R measurement in these diseases, except perhaps in HIV infection.

In infectious mononucleosis[59,60,149] analysis of serum sIL-2R demonstrated significantly elevated levels as compared to normal controls. Increased levels of sIL-2R were correlated with increased percentages of activated CD8+ T-cells. These values tend to decrease progressively in relation to the reduction of activated CD8+ and symptom relief. Patients with X-linked lymphoproliferative syndrome and virus-associated haemophagocytic syndrome, two syndromes associated with severe acute Epstein Barr virus infections, demonstrated the most dramatic increase in sIL-2R levels.

During measles,[150] the levels of serum sIL-2R increase before the onset of the rash and remain elevated for at least 4 weeks. Peak numbers of peripheral blood IL-2R-expressing lymphocytes appear coincidentally with the onset of the rash and remain for approximately 10 days.

In hepatitis B virus[151–153] infection increased values of sIL-2R have been found during both acute liver damage and active chronic phase. Serum sIL-2R has been measured in patients with acute type B hepatitis, patients with chronic type B hepatitis and controls. All patients with acute type B hepatitis presented levels significantly higher than those of normal controls or of patients with chronic type B hepatitis. Serial follow-up showed that serum levels of sIL-2R tended to return to normal 2–4 months after onset of acute hepatitis along with the normalization of alanine aminotransferase. Patients with chronic type B hepatitis also had significantly higher levels of sIL-2R that varied considerably with liver flogosis, i.e. significantly lower levels were detected in patients with chronic infection who had no evidence of active liver disease. In chronic infection, in response to therapy with prednisone and/or interferon, serum sIL-2R fell significantly and a significant correlation between serum sIL-2R and alanine amino transferase levels has been observed. High sIL-2R levels have also been observed during hepatitis A infection, while lower values have been seen during hepatitis C infection.[151]

Many reports show that serum sIL-2R levels are increased in human immunodeficiency virus (HIV) seropositive and AIDS-affected[50,154–164] subjects and are inversely correlated with both relative and absolute numbers of CD4+ T-cells and with the CD4/CD8 ratio, although it has to be pointed out that this relation probably takes place only late after seroconversion. Soluble IL-2R levels of seropositive subjects have been demonstrated to be predictive for development of AIDS, to correlate with response to therapy and to increase as a direct effect of HIV infection and not only as a consequence of opportunistic infections. Finally, serum sIL-2R does not correlate with either other serum markers of HIV infection (i.e. neopterin and $\beta_2$-microglobulin) or cell membrane expression of CD25, that is decreased in HIV infection. In one study, however, a significant association between blood sIL-2R and neopterin levels has been observed.

A significant increase of serum sIL-2R levels, which reflects chronic activation of the immune system, has been demonstrated in patients with systemic parasitic diseases. In fact, elevated sIL-2R levels has been observed in the serum of patients with plasmodium falciparum infection, chronic hepatosplenic schistosomiasis, strongyloidosis, fasciolosis, alveolar echinococcosis of the liver and visceral leishmaniasis.[165–168] Conversely, this parameter is not significantly increased in localized parasitic disease, particularly in intestinal schistosomiasis. The circulating levels of sIL-2R appear to reflect the extent as well as the severity of the diseases. It has been suggested that in alveolar echinococcosis of the liver and in chronic hepatosplenic schistosomiasis, both characterized by the development of liver granulomas, sIL-2R could be released by activated macrophages rather than by T-lymphocytes. Incidentally, as discussed by Bresson-Hadni *et al.*[166] this might also be true for other granulomatous diseases such as sarcoidosis. Sera from patients with visceral leishmaniasis at the moment of the diagnosis, during the course of the disease and after clinical recovery, have been analysed for the concentration of serum sIL-2R. The results show that sIL-2R is a marker of disease activity, since it is in high concentration at the beginning of infection and returns to the normal range following successful chemotherapy. At the same time as serum analysis for sIL-2R, MNCs of patients were stimulated with PHA or antigen and supernatant tested for IL-2 and IFN-τ production. Data demonstrate that there is an inverse relation between concentration of IL-2 and IFN-τ in the supernatants and sIL-2R secretion in the sera.

In leprosy[169,170] a moderate increase of serum sIL-2R levels has been found in untreated lepromatous patients, as opposed to the decreased values found in tuberculoid patients. Highly elevated levels were associated with reversal reaction and, to a lesser extent, with erythema nodosum leprosum. Thus, the potential for developing DTH reaction to M. Leprae antigens is not sufficient to induce elevated serum sIL-2R levels by itself. The high serum sIL-2R levels found in patients with reversal reaction instead provides indirect evidence for the association of elevated sIL-2R with accelerated or profound DTH reaction. Treatment with corticosteroids was invariably associated with a concurrent drop in serum sIL-2R levels, thus providing an objective measure of compliance. During erythema nodosum there is a transient rise in agalactosyl IgG and this increase parallels an increase in blood sIL-2R. Finally, increased levels of sIL-2R have been observed in patients affected by tuberculosis.[171]

### sIL-2R Release in Transplanted Patients

Activation of T-lymphocytes in response to alloantigens is a central component of the rejection process after organ transplantation. Thus, in the absence of infection, one could assume that increased sIL-2R levels might be a tool to evaluate the presence of rejection activity.[172]

Indeed, as regards sIL-2R production in kidney[173–176] recipients, it has been demonstrated that serum levels of sIL-2R are significantly higher in patients suffering renal allograft rejection as compared to patients with stable graft function and that the serial evaluation of serum sIL-2R increases the specificity and sensitivity of the test. Furthermore, it has been observed that during rejection episodes urine sIL-2R levels are increased in a pattern undistinguishable from those of serum levels. The increase of serum sIL-2R has also been shown to be comparable to the rise in serum creatinine values which is observed in rejection episodes, the predictive value of the combined tests being superior to either alone. Moreover, it has been observed that the raise in serum sIL-2R levels is higher during rejection episodes than in the case of other forms of allograft dysfunction, e.g. infections or cyclosporin A (CsA) toxicity. In fact nephrotoxicity as a result of CsA treatment has been shown to be a cause of a slight elevation in SIL-2R levels. However, concerning infectious diseases, patients with cytomegalovirus infections have been shown to have sIL-2R levels equivalent to those found in patients undergoing rejection. Again, the combination of sIL-2R and creatinine assays greatly enhances the predictive value of either test alone for distinguishing acute graft rejection from infection. However, the administration of anti-T-cell antibodies to patients with kidney allograft rejection is followed by a rise in plasma sIL-2R.

Recently, the clinical utility of monitoring sIL-2R levels in renal transplant recipients has been appraised by a multicentre study. A significant increase of serum sIL-2R levels was observed in the presence of rejection episodes as compared to prerejection values and to values observed in stable patients, respectively. Moreover, sIL-2R concentrations were significantly higher in cadaver recipients than in living renal donor recipients, not only at the time of rejection, but also prior to rejection and early posttransplantation. Finally, sIL-2R levels may also be influenced by therapy, since patients who received antilymphocyte antibody induction therapy showed higher values.[177]

Both serum and biliary sIL-2R levels are significantly higher in patients undergoing acute as well as chronic liver[178–180] rejection as compared to the control group. Serum and biliary sIL-2R increase have been shown to occur before the diagnosis of rejection was possible based on clinical symptoms and conventional laboratory tests, but biliary values appeared to be more specific and sensitive for prediction of graft outcome, although serum sIL-2R rises earlier. Biliary sIL-2R levels rose 24 h after serum levels, suggesting that lymphocyte activation occurs outside the graft and then activated cells localize to the graft. Although infections may also cause an increase in serum sIL-2R levels, these have always been shown to be lower than those observed in rejection episodes. Finally, it has been shown that biliary and serum sIL-2R levels are significantly lower in patients with complications other than acute graft rejection and in patients undergoing chronic rejection and that serum sIL-2R levels are inversely related to the duration of the disease.

Experience with sIL-2R assays in heart and lung[181–184] transplant patients is limited. Single determinations of sIL-2R levels in both serum and plasma have instead not proved to be useful in detecting heart transplantation rejection. In fact no correlation has been found between sIL-2R and histological findings of graft rejection in endomyocardial biopsies. When serial measurements of serum sIL-2R were performed, a significant difference was observed only when mean sIL-2R levels of patients presenting a severe rejection were compared to sIL-2R levels from all other patients. However, an increase of sIL-2R shortly after heart transplantation has been shown to correlate with the development of coronary arteriopathy and with mortality during long-term follow-up. Finally, a correlation has been observed between serum sIL-2R levels and graft rejection after heart–lung and lung transplants. In fact, the relative sIL-2R levels in patients with heart–lung and lung transplants could help to differentiate between infection and rejection. In a prospective blind study sIL-2R was markedly elevated during rejection. Interestingly, sIL-2R levels seemed to be related to the quantity of allograft tissue, with mean sIL-2R levels during rejection episodes in single-lung recipients being roughly half the level seen in the recipients of two lungs (bilateral lung or heart–lung transplants).

Although these results are suggestive, more clinical experience with sIL-2R is needed to make a final statement on the utility of sIL-2R as a rejection marker at least in liver, heart and lung transplants.

### sIL-2R Production in Other Diseases

Following mytogen stimulation, lymphocytes from immunosuppressed burn[185,186] patients exhibit a reduced number of CD25 positive cells. In contrast, sera from burn patients contain markedly elevated levels of sIL-2R that are inversely proportional to the density of CD25 antigens expressed on the surface of mytogen-activated lymphocytes. In recovering subjects a progressive reduction of serum sIL-2R, which paralleled the restoration of lymphocyte reactivity to mytogen stimulation, has been observed. Soluble IL-2R was significantly higher in sera from burn patients who did not survive than in sera from patients who survived. Throughout the postburn period a significant proportion of patients studied also demonstrated increasing levels of serum IL-2. In this period *in vitro* IL-2 production and sIL-2R secretion in patient cultures were significantly reduced as compared to the controls. These observations suggest that in the burn patients altered synthesis and/or secretion of the soluble form of sIL-2R may be regulated to IL-2 content.

In sera from patients with common variable immunodeficiency (CVI)[187] levels of soluble CD8, soluble CD25 and $\beta_2$-microglobulin were raised significantly above levels in normal sera. They correlated with the extent of the defects in the B-lymphocytes assessed *in vitro*, as well as with the clinical severity of the disease. The selective release of these molecules into sera may indicate that abnormal cellular activation occurs in most CVI patients.

Patients on chronic haemodialysis[188] had increased plasma sIL-2R. The reduced kidney excretion in chronic renal failure could be a simple explanation. Haemodialysis patients present lymphopenia and higher CD4/CD8 ratios, CD16 counts and sIL-2R concentrations as compared to controls. A significant inverse correlation was found between sIL-2R concentration and lymphocyte count and between sIL-2R concentration and CD4/CD8 ratio. An increase of sIL-2R concentration due to abnormal T-cell preactivation in haemodialysis patients with non-reused cuprophan membranes could perhaps contribute to cell immunity impairment through IL-2 binding and inhibition of T-cell activation.

Soluble IL-2R has also been measured in the serum of patients with liver cirrhosis, patients with obstructive jaundice,[189] patients with alcoholic liver disease without evidence of cirrhosis, healthy persons and patients with unrelated diseases. In patients with cirrhosis and obstructive jaundice sIL-2R was significantly increased as compared to healthy subjects and patients with unrelated diseases. No difference was found between patients with cirrhosis due to alcohol abuse and chronic hepatitis B. In obstructive jaundice, sIL-2R correlated with alkaline phosphatase as a marker of cholestasis. These data show that in spite of the apparent depressed cellular immune defence, both in liver cirrhosis and obstructive jaundice, there is a general activation of the immune system.

### Concluding Remarks

A number of receptors for growth factors and differentiation antigens have been found to be released or secreted by different cells. Little is known, however, about the function of these soluble molecules during cell growth and differentiation. Although investigations of cytokine-mediated growth effects have focused almost exclusively on the interaction of molecules at the cell surface, it is possible that released molecules that can potentially bind ligands and/or act to transmit important signals between cells might function in promoting or regulating these processes.[44,114–116,118,133,190] In this regard, as stated earlier, it is of note that the released form of IL-2R, which is 10 kDa smaller due to lack of transmembrane and intracytoplasmic domains, still retains the ability to bind IL-2 with an affinity similar to the membrane-bound form of the Tac molecule, although it is approximately 100- to 1 000-fold lower than the high-affinity IL-2R complex. Indeed, as discussed in the present review, experiments examining the addition of exogenous sIL-2R to *in vitro* IL-2 functional assays show inhibition of the stimulatory effects of IL-2. Further, immunologic assessment of patients after therapeutic infusion of high doses of IL-2, which is invariably accompanied by a marked increase in serum sIL-2R levels, reveals a decreased responsiveness *in vivo* as well as *in vitro* to recall antigens.[90,109,191] The ability of this molecule to bind to IL-2 suggests then a potential role in the regulation of IL-2-dependent cell function by competing with cellular IL-2R for the growth factor IL-2, thus down-regulating the immune response. However, it has to be stressed that these findings may be due to the use of supraphysiological doses of sIL-2R and IL-2, respectively, such as that currently used in *in vitro* studies and for therapeutic purposes. This may activate negative feedback circuits, thus explaining the functional defect observed.

Alternatively, the rapid increase in sIL-2R after cellular activation has occurred, may represent the means, by a proteolytic cleavage mechanism, for activated cells to reduce surface receptor density below a critical threshold level, thus preventing an ongoing, perhaps undesirable, response. Another possibility is that sIL-2R acts as a binding protein, effectively prolonging the half-life of IL-2, with low-affinity sIL-2R delivering IL-2 to high-affinity membrane receptors and/or serving to locally confine the effects of IL-2, hence, keeping immune responses local.[42,44] Finally, it should not be forgotten that the presence of sIL-2R in the serum might be an epiphenomenon simply reflecting the activation of the immune system. Indeed, we have observed that, after MNC stimulation with PHA, there is a significant correlation between the levels of sIL-2R in supernatants at 24 h and the proliferative response at 48 h (Fig. 1). We can

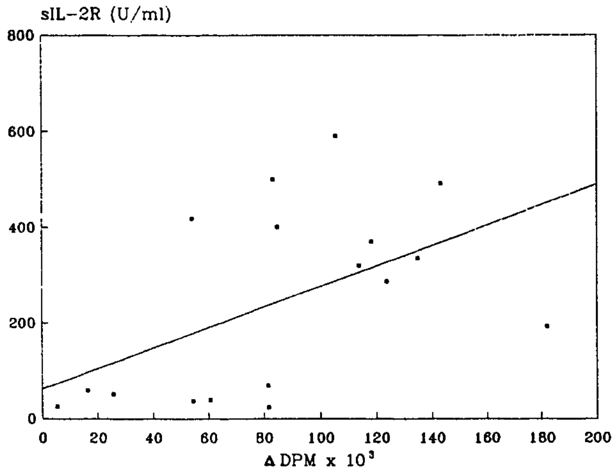

FIG. 1. Correlation between sIL-2R levels measured at 24 h of culture and proliferative response to PHA determined at 48 h of culture ($r = 0.52$, $p = 0.03$). MNCs from 16 healthy subjects were divided into two aliquots. The first one was processed to produce sIL-2R, the second one was stimulated for 48 h with PHA and proliferation assayed. Experiments were performed as previously described.[147] Soluble IL-2R values are expressed as unit/ml. Proliferative responses are expressed as mean Δdpm which is equal to the dpm of the cells in the mitogen-containing medium minus the dpm of the cells in the medium without mitogens.

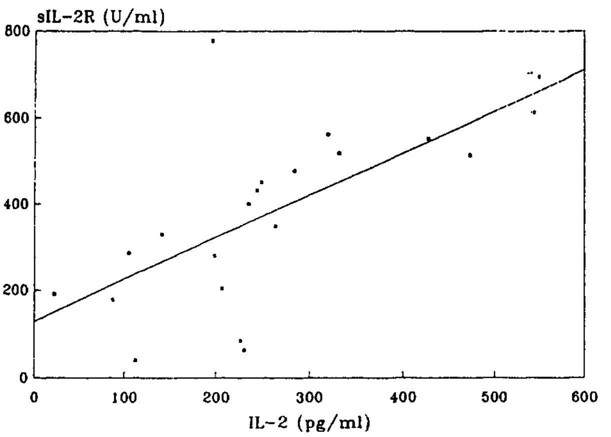

FIG. 2. Correlation between sIL-2R (U/ml) and IL-2 (pg/ml)levels in cultures from 20 healthy subjects ($r = 0.67$, $p = 0.0009$). Experiments were performed as previously described.[146,147] IL-2 values were quantified by commercial ELISA.

conclude that sIL-2R does not impair MNC mitogen responsiveness, at least at physiological concentrations *in vitro*. More probably sIL-2R release is expression of the IL-2 driven MNC activation, as depicted in Fig. 2, which shows that there is a linear correlation between IL-2 and sIL-2R production at 24 h by *in vitro* PHA-stimulated MNCs.

However, the blood sIL-2R levels depend on the number of producing cells and on the number of molecules per cell and therefore sIL-2R blood values represent an index of the number and functional state of producing cells, although in some conditions no correlation has been found between IL-2R expression by circulating cells (as detected by cytometric techniques) and sIL-2R serum levels. This discrepancy may reflect the different dynamics of the two forms of the receptor or, most probably, it is due to the local production of sIL-2R which may constitute an important source of serum sIL-2R.[172] Moreover, metabolic alterations, particularly impairments of renal function, can affect the level of serum sIL-2R. In fact, impaired renal function elevates serum sIL-2R, as this molecule is likely to be actively transported into the urine.[56,58] The serum sIL-2R level then is a sensitive and quantitative marker of circulating MNC activation and may also reflect immune activation in other tissues or fluid compartments. The basal serum sIL-2R levels may therefore be considered to be a reflection of the baseline level of immune activation under normal physiologic stimuli and immunological monitoring of the immune system, mainly of T-cell activation (but also of monocytes in granulomatous disorders), might be the target for the measurement of sIL-2R.

On the other hand, the increased concentration of sIL-2R observed in lymphoid malignancies appears to reflect tumour burden, so that measurement of sIL-2R serum levels has been proved to be helpful in estimating prognosis and diagnosing relapse. In other diseases like allograft rejection, infections, autoimmune diseases and non-haematological malignancies, the elevation of sIL-2R is likely to indicate the immune system activation in response to antigenic challenge by infectious agents, autoantigens and cross-reacting determinants and malignant cell antigens, respectively and, therefore, it can be successfully used to monitor disease progress and therapy effects.

Finally, it is puzzling that in many conditions in which an increase of blood sIL-2R values has been observed, MNCs display a decreased *in vitro* capacity to produce sIL-2R. This is indeed the case in autoimmune diseases tested in this regard, burns, some kind of malignancies, as well as in healthy elderly subjects and young healthy HLA-B8,DR3 positive individuals.[98,122,146,147,185,186]

However, in these conditions, more complex abnormalities of the IL-2/IL-2R system may be described, consisting of a defect in IL-2 release by mitogen-activated mononuclear cells, increased expression of CD25 in unstimulated peripheral blood lymphocytes from patients with active disease, low expression of C25 in mitogen-stimulated cells from the same patients, and high serum levels of IL-2 correlating with some indices of activity.[10,98,122,146,147,185,186,192]

As IL-2 induces soluble and membrane-bound IL-2Rα both *in vitro* and *in vivo*, it cannot be ruled out that the observed changes in sIL-2R levels are secondary to the changes in IL-2 concentration and/or production (Fig. 2).[100,101]

Concerning the defective *in vitro* IL-2 production it has been suggested that precommitment and preactivation of peripheral T-cells *in vivo* result in a transient exhaustion of IL-2 secretion. As an alternative to the exhaustion theory, it is conceivable that production of IL-2 by a subpopulation of *in vivo*-activated lymphocytes elicits feedback regulation pathways that attempt to restore the homeostasis of the IL-2/IL-2R system by non-specifically suppressing IL-2 production.[10]

However, our studies on immune function of HLA-B8,DR3 positive and elderly individuals suggest that these two groups of subjects are low responders. In fact findings such as high serum sIL-2R levels and an increased number of CD25+ circulating lymphocytes which may be suggestive of a high responder status, are instead an expression of the relative inability of these subjects to remove immunological stimuli from the body. This, in our opinion, is responsible for a prolonged stimulation and may lead to the persistence of signs of immune system activation at least in these subjects and in autoimmune patients.[146,147,192–194]

### References

1. Morgan RA, Ruscetti FW, Gallo RC. Selective *in vitro* growth of T lymphocytes. *Science* 1976; **193**: 1007–1008.
2. Smith KA. Interleukin-2: inception, impact and implications. *Science* 1988; **240**: 1169–1176.
3. Taniguchi T, Matsui H, Fujita T, *et al.* Structure and expression of cloned cDNA for human interleukin-2. *Nature* 1983; **302**: 305–310.
4. Smith K. Interleukin-2. *Annu Rev Immunol* 1984; **2**: 319–333.
5. Mosmann TR, Coffman RL. Heterogeneity of cytokine secretion patterns and function of helper T cells. *Adv Immuol* 1989; **46**: 11–147.
6. Mosmann TR, Coffman RL. Different patterns of lymphokine secretion lead to different functional properties. *Annu Rev Immunol* 1989; **7**: 145–173.
7. Romagnani S. Type 1 T helper and Type 2 T helper cells: functions, regulation and role in protection and disease. *Int J Clin Lab Res* 1991; **21**: 152–158.
8. Moller G. Activation antigens and signal transduction in lympohocyte activation. *Immunol Rev* 1987; **95**: 1–194.
9. Ullman K, Northrop JP, Verweij CL, Crabtree GR. Transmission of signals from T lymphocyte antigen receptor to the genes responsible for cell proliferation and immune function. *Annu Rev Immunol* 1990; **8**: 421–452.
10. Kroemer G, Andreu JL, Gonzalo JA, Gutierrez-Ramos JC, Martinez AC. Interleukin-2, autotolerance, and autoimmunity. *Adv Immunol* 1991; **50**: 147–235.
11. Greene WC, Leonard WJ. The human interleukin-2 receptor. *Annu Rev Immunol* 1986; **4**: 69–95.
12. Greene WC. Interleukin-2 receptor. In: Roitt IM, Delves PJ, eds. *Encyclopedia of Immunology*. San Diego: Academic Press, 1992; 906–908.
13. Greene WC. The human interleukin 2 receptor: a molecular and biochemical analysis of structure and function. *Clin Res* 1987; **35**: 439–450.
14. Waldmann TA. The interleukin-2 receptor. *J Biol Chem* 1991; **266**: 2681–2684.
15. Waldmann TA. The structure and expression of interleukin-2 receptors on normal and malignant lymphocytes. *Science* 1986; **232**: 727–732.
16. Leonard WJ, Depper JM, Crabtree GR, *et al.* Molecular cloning and expression of cDNAs for the human interleukin-2 receptor. *Nature* 1984; **311**: 626–631.
17. Nikaido T, Shimuzu A, Ishida N, *et al.* Molecular cloning of cDNA encoding human interleukin-2 receptor. *Nature* 1984; **311**: 631–636.
18. Cosman D, Ceretti DP, Larsen A, *et al.* Cloning, sequence and expression of human interleukin-2 receptor. *Nature* 1984; **312**: 768–771.
19. Hatakeyama M, Tsudo M, Minamoto S, *et al.* Interleukin-2 receptor $\beta$ chain gene: generation of three receptor forms by cloned human $\alpha$ and $\beta$ chain cDNAs. *Science* 1989; **244**: 551–560.
20. Leonard WJ, Depper JM, Robb RJ, Waldmann TA, Greene WC. Characterization of the human receptor for T cell growth factor. *Proc Natl Acad Sci USA* 1983; **80**: 6957–6961.
21. Uchiyama T, Broder S, Waldmann TA. A monoclonal antibody (anti-Tac) reactive with activated and functionally mature human T cells. I. Production of anti-Tac monoclonal antibody and distribution of Tac (+) cells. *J Immunol* 1981; **126**: 1393–1397.
22. Dower SK, Smith CA, Park LS. Human cytokine receptors. *J Clin Immunol* 1990; **10**: 289–299.
23. Miyajima A, Kitamura T, Harada N, Yokota T, Arai K. Cytokine receptors and signal transduction. *Annu Rev Immunol* 1992; **10**: 295–331.
24. Wang HM, Smith KA. The interleukin 2 receptor. Functional consequences of its bimolecular structure. *J Exp Med* 1987; **166**: 1055–1069.
25. Teshigawara K, Wang HM, Kato K, Smith KA. Interleukin 2 high-affinity receptor expression requires two distinct binding proteins. *J Exp Med* 1987; **165**: 223–238.
26. Robb RJ, Rusk CM, Yodor J, Greene WC. Interleukin 2 binding molecule distinct from the Tac antigen: analysis of its role in formation of high-affinity receptors. *Proc Natl Acad Sci USA* 1987; **84**: 2002–2006.
27. Robb RJ, Greene WC. Internalization of interleukin 2 is mediated by the $\beta$ chain of the high-affinity interleukin 2 receptor. *J Exp Med* 1987; **165**: 1201–1206.
28. Siegel JP, Sharon M, Smith PL, Leonard WJ. The IL-2 receptor $\beta$ chain (p70): role in mediating signals for LAK, NK, and proliferative activities. *Science* 1987; **238**: 75–78.
29. Takeshita T, Asao H, Ohtani K, *et al.* Cloning of the $\tau$ chain of the human IL-2 receptor. *Science* 1992; **257**: 379–382.
30. Hatakeyama M, Kona T, Kobayashi N, *et al.* Interaction of the IL-2 receptor with the src-family kinase $p56^{lck}$: identification of novel intermolecular association. *Science* 1991; **252**: 1523–1528.
31. Taga K, Kasahara Y, Yachie A, Miyawaki T, Taniguchi N. Preferential expression of IL-2 receptor subunits on memory populations within $CD4^+$ and $CD8^+$ T cells. *Immunology* 1991; **72**: 14–19.
32. Herrman F, Cannistra SA, Levine H, Griffin JD. Expression of interleukin 2 receptor and binding of interleukin 2 by gamma interferon-induced human leukemic and normal monocytic cells. *J Exp Med* 1985; **162**: 1111–1116.
33. Waldmann TA, Goldman CK, Robb RJ, *et al.* Expression of interleukin 2 receptors on activated human B cells. *J Exp Med* 1984; **160**: 1450–1466.
34. Jung LK, Hara T, Fu SM. Detection and functional studies of p60–65 (Tac antigen) on activated human B cells. *J Exp Med* 1984; **160**: 1597–1602.
35. Holter W, Goldman CK, Casabo L, Nelson DL, Greene WC, Waldmann TA. Expression of functional IL 2 receptors by lipopolysaccharide and interferon-$\tau$ stimulated human monocytes. *J Immunol* 1987; **138**: 2917–2922.
36. Kniep EM, Strelow I, Lohmann-Matthes ML. The monocyte interleukin-2 receptor light chain production of cell-associated and soluble interleukin-2 receptor by monocytes. *Immunology* 1992; **75**: 299–304.
37. Pizzolo G, Chilosi M, Semenzato G, *et al.* Immunohistological analysis of Tac antigen expression in tissues involved by Hodgkin's disease. *Br J Cancer* 1984; **50**: 415–417.
38. Hsu SM, Young K, Jaffe ES. Phenotypic expression of Hodgkin's and Reed–Sternberg cells in Hodgkin's disease. *Am J Pathol* 1985; **118**: 209–217.
39. Strauchen JA, Breakstone BA. IL-2 receptor expression in human lymphoid lesions: immunohistochemical study of 166 cases. *Am J Pathol* 1987; **126**: 506–512.
40. Sheibani K, Winberg CD, Van de Velde S, Bl Blayney DW, Rappaport H. Distribution of lymphocytes with interleukin 2 receptors (Tac antigens) in reactive lymphoproliferative processes, Hodgkin's disease and non-Hodgkin's lymphomas: an immunohistologic study of 300 cases. *Am J Pathol* 1987; **127**: 27–37.
41. Yodoi J, Uchiyama T. Diseases associated with HTLV-1: virus, IL-2 receptor dysregulation and redox regulation. *Immunol Today* 1992; **13**: 405–410.
42. Rubin LA, Kurman CC, Fritz ME, *et al.* Soluble interleukin 2 receptors are released from activated human lymphoid cells *in vitro*. *J Immunol* 1985; **135**: 3172–3177.
43. Rubin LA, Kurman CC, Biddison WE, Goldman ND, Nelson DL. Monoclonal antibody 7G7/B6, binds to an epitope on the human interleukin-2 (IL-2) receptor that is distinct from that recognized by IL-2 or anti-Tac. *Hybridoma* 1985; **4**: 91–102.
44. Rubin LA, Jay G, Nelson DL. The released interleukin 2 receptor binds interleukin 2 efficiently. *J Immunol* 1986; **137**: 381–384.
45. Treiger B, Leonard WJ, Svetlik P, Rubin LA, Nelson DL, Greene WC. A secreted form of the IL-2 receptor encoded by an "anchor minus" cDNA mutant. *J Immunol* 1986; **136**: 4099–4105.
46. Nelson DL, Rubin LA, Kurman CC, Fritz ME, Boutin B. An analysis of the cellular requirements for the production of soluble interleukin-2 receptors *in vitro*. *J Clin Immunol* 1986; **6**: 114–120.
47. Nelson DL, Kurman CC, Fritz ME, Boutin B, Rubin LA. The production of soluble and cellular interleukin-2 receptors by cord blood mononuclear cells following *in vitro* activation. *Pediatr Res* 1986; **20**: 136–139.
48. Lai KN, Leung JC, Lai FM. Soluble interleukin-2 receptor release, interleukin-2 production and interleukin-2 receptor expression in activated T lymphocytes *in vitro*. *Pathology* 1991; **23**: 224–228.
49. Rubin LA, Nelson DL. The soluble interleukin-2 receptor: biology, function, and clinical application. *Ann Int Med* 1990; **113**: 619–627.
50. Kloster BE, John PA, Miller LE, *et al.* Soluble interleukin 2 receptors are elevated in patients with AIDS or at risk of developing AIDS. *Clin Immunol Immunopathol* 1987; **45**: 440–446.
51. Komp DM, Shapiro E, McNamara J. Soluble interleukin-2 receptor in childhood non-Hodgkin's lymphoma. *Blood* 1988; **71**: 1172–1173.
52. Manoussakis MN, Stavropoulos ED, Germanidis GS, *et al.* Soluble interleukin-2 receptors and autoantibodies in the serum of healthy elderly individuals. *Autoimmunity* 1990; **7**: 129–137.
53. Giannitsisis DJ, Muller-Hof B, Hacker-Shahin B. Serum soluble interleukin-2 receptor levels of normal blood donors. *Cytobios* 1991; **68**: 161–164.
54. Lemmer B, Schwulera U, Thrun A, Lissner R. Circadian rhythm of soluble interleukin-2 receptor in healthy individuals. *Eur Cytokine Netw* 1992; **3**: 335–336.
55. Famularo G, Giacomelli R, Sacchetti S, Luciani AM, Tonietti G. The soluble interleukin-2 receptor: biological and clinical relevance. *EOS* 1990; **10**: 8–14.
56. Marcon L, Fritz ME, Kurman CC, Jensen JC, Nelson DL. Soluble Tac peptide is present in the urine of normal individuals and at elevated levels in patients with adult T cell leukaemia (ATL). *Clin Exp Immunol* 1988; **73**: 29–33.
57. Lovett DH, Ryan JL, Sterzel RB. A thymocyte-activating factor derived from glomerular mesangial cells. *J Immunol* 1983; **130**: 1796–1801.
58. Novick D, Engelmann H, Wallach D, Rubistein M. Soluble cytokine receptors are present in normal human urine. *J Exp Med* 1989; **170**: 1409–1414.
59. Pizzolo G, Chilosi M, Semenzato G. The soluble interleukin-2 receptor in haematological disorders. *Br J Haematol* 1987; **67**: 377–380.
60. Pizzolo G. The soluble interleukin-2 receptor as a new biological marker in disease. *Immunol Clin* 1988; **7**: 13–21.
61. Pui CH. Serum interleukin-2 receptor: clinical and biological implications. *Leukaemia* 1989; **3**: 323–327.
62. Jacques Y, Le Mauff B, Boeffard F, Godard A, Soulillou JP. A soluble interleukin 2 receptor produced by a normal alloreactive human T cell clone binds interleukin 2 with low affinity. *J Immunol* 1987; **139**: 2308–2316.
63. Worman CP, Cawley JC. Monoclonal antibody-defined T-cell subsets in hairy-cell leukaemia. *Scand J Haematol* 1982; **29**: 338–344.
64. Korsmeyer SJ, Greene WC, Cossman J, *et al.* Rearrangement and expression of immunoglobulin genes and expression of Tac antigen in hairy cell leukaemia. *Proc Natl Acad Sci USA* 1983; **80**: 4522–4526.
65. Chilosi M, Semenzato G, Cetto G, *et al.* Soluble interleukin-2 receptors in the sera of patients with hairy cell leukaemia: relationship with the effect of recombinant $\alpha$-interferon therapy on clinical parameters and natural killer *in vitro* activity. *Blood* 1987; **70**: 1530–1535.

66. Steis RG, Marcon L, Clark J, *et al.* Serum soluble IL-2 receptor as a tumor marker in patients with hairy cell leukemia. *Blood* 1988; **71**: 1304–1309.
67. Waldmann TA, Greene WC, Sarin TS, *et al.* Functional and phenotypic comparison of human T cell leukemia/lymphoma virus positive adult T cell leukemia with human T cell leukemia/lymphoma virus negative Sezary leukemia, and their distinction using anti-Tac. Monoclonal antibody identifying the human receptor for T cell growth factor. *J Clin Invest* 1984; **73**: 1711–1718.
68. Tamura K, Nagamine N, Araki Y, *et al.* Clinical analysis of 33 patients with adult T-cell leukemia (ATL)-diagnostic criteria and significance of high- and low-risk ATL. *Int J Cancer* 1986; **37**: 335–341.
69. Greene WC, Leonard WJ, Depper JM, Nelson DL, Waldmann TA. The human interleukin-2 receptor: normal and abnormal expression in T cells and leukemias induced by the human T-lymphotropic retroviruses. *Ann Int Med* 1986; **105**: 560–572.
70. Yasuda N, Lai PK, Ip SH, *et al.* Soluble interleukin 2 receptors in sera of Japanese patients with adult T cell leukemia mark activity of disease. *Blood* 1988; **71**: 1021–1026.
71. Marcon L, Rubin LA, Kurman CC, *et al.* Elevated serum levels of soluble Tac peptide in adult T-cell leukemia: correlation with clinical status during chemotherapy. *Ann Int Med* 1988; **109**: 274–279.
72. Foa R, Giovarelli M, Jemma C, *et al.* Interleukin-2 (IL-2) and interferon-τ production by T lymphocytes from patients with B-chronic lymphocytic leukemia: evidence that normally released IL-2 is absorbed by the neoplastic B cell population. *Blood* 1985; **66**: 614–619.
73. Semenzato G, Foa R, Agostini C, *et al.* High serum levels of soluble interleukin receptor in patients with B chronic lymphocytic leukemia. *Blood* 1987; **70**: 396–400.
74. Kay NE, Burton J, Wagner D, Nelson DL. The malignant B cells from B-chronic lymphocytic leukemia patients with release Tac-soluble interleukin 2 receptors. *Blood* 1988; **72**: 447–450.
75. Touw I, Delwel R, Bolhuis R, van Zanen G, Lowenberg B. Common and pre-B acute lymphoblastic leukemia cell express interleukin 2 receptors, and interleukin 2 stimulates *in vitro* colon formation. *Blood* 1985; **66**: 556–561.
76. Matsuoka M, Hattori T, Kawano F, Ishii T, Uchiyama T, Takatsuki K. Expression of Tac antigen on human immature B-cell lineage leukemic cells. *Leuk Res* 1986; **10**: 597–603.
77. Morishima Y, Morishita Y, Adachi K, Tanimoto M, Ohno R, Saito H. Phorbol ester induces interleukin-2 receptor on the cell surface of precursor thymocyte leukemia with no rearrangement of T cell receptor β and τ genes. *Blood* 1987; **70**: 1291–1296.
78. Wormann B, Anderson JM, Ling ZD, LeBien TW. Structure/function analyses of IL-2 binding proteins on human B cell precursor acute lymphoblastic leukemias. *Leukaemia* 1987; **1**: 660–666.
79. Pui CH, Ip SH, Iflah S, *et al.* Serum interleukin 2 receptor levels in childhood acute lymphoblastic leukemia. *Blood* 1988; **71**: 1135–1137.
80. Pui CH, Ip SH, Kung P, *et al.* High serum interleukin-2 receptor levels are related to advanced disease and a poor outcome in childhood non-Hodgkin's lymphoma. *Blood* 1987; **70**: 624–628.
81. Wagner DK, Kiwanuka J, Edwards BK, Rubin LA, Nelson DL, Magrath IT. Soluble interleukin-2 receptor levels in patients with undifferentiated and lymphoblastic lymphomas: correlation with survival. *J Clin Oncol* 1987; **70**: 1262–1274.
82. Harrington DS, Patil K, Lai PK, *et al.* Soluble interleukin 2 receptors in patients with malignant lymphoma. *Arch Pathol Lab Med* 1988; **112**: 597–601.
83. Pizzolo G, Chilosi M, Vinante F, *et al.* Soluble interleukin-2 receptors in the serum of patients with Hodgkin's disease. *Br J Cancer* 1987; **55**: 427–428.
84. Pui CH, Ip SH, Thompson E, *et al.* High serum interleukin-2 receptor (IL2R) levels correlate with a poor prognosis in childhood Hodgkin's disease. *Proc 20th Int Soc Pediatr Oncol* 1988: 150–153.
85. Caruso C, Modica MA, Candore G, *et al.* Soluble interleukin-2 receptor *in vitro* production by mononuclear cells from Hodgkin patients. *Boll Ist Sieroter Milan* 1990; **69**: 335–338.
86. Zwierzina H, Herold M, Schollenberger S, Geissler D, Schmalzl F. Detection of soluble interleukin-2 receptor in the serum of patients with myelodysplastic syndromes: induction and therapy with GM-CSF. *Br J Haematol* 1991; **79**: 438–443.
87. Nielsen H, Nielsen HJ, Tuede N, *et al.* Immune dysfunction in multiple myeloma. Reduced natural killer cell activity and increased levels of soluble interleukin-2 receptors. *Apmis* 1991; **99**: 340–346.
88. Gilmore SJ, Benson EM, Kelly JW. Interleukin-2 receptor levels and cutaneous T cell lymphoma. *Austral J Dermatol* 1991; **32**: 101–105.
89. Zachariae C, Larsen CS, Kaltoft K, Deleuran B, Larsen CG, Thestrup-Pedersen K. Soluble interleukin-2 receptor serum levels and epidermal cytokines in mycosis fungoides and related disorders. *Acta Dermatol Venereol* 1991; **71**: 465–470.
90. Dummer R, Posseckert G, Nestle F, *et al.* Soluble interleukin-2 receptors inhibit interleukin-2 dependent proliferation and cytotoxicity: explanation for diminished natural killer cell activity in cutaneous T cell lymphomas *in vivo*? *J Invest Dermatol* 1992; **98**: 50–54.
91. Visani G, Delwel R, Touw I, Bot F, Lowenberg B. Membrane receptors for interleukin 2 on hematopoietic precursors in chronic myeloid leukemia. *Blood* 1987; **69**: 1187–1192.
92. Motoi T, Uchiyama T, Hori T, Itoh K, Uchinhinhino H, Ueda R. Elevated serum soluble interleukin-2 receptor (Tac antigen) levels in chronic myelogenous leukaemia patients with blastic crisis. *Blood* 1989; **74**: 1052–1057.
93. Zerler B. The soluble interleukin-2 receptor as a marker for human neoplasia and immune status. *Cancer Cell* 1991; **3**: 471–479.
94. Marino P, Cugno M, Preatoni A, *et al.* Increased levels of soluble interleukin-2 receptors in serum of patients with lung cancer. *Br J Cancer* 1990; **61**: 434–435.
95. Poulakis N, Sarandakou A, Rizos D, Phocas I, Kontozoglou T, Polyzogopoulos D. Soluble interleukin-2 receptors and other markers in primary lung cancer. *Cancer* 1991; **68**: 1045–1049.
96. Tisi E, Lissoni P, Angeli M, Arrigoni C, Corno E, Cassina E, Ballabio E, Benenti C, Barni S, Tancini G. Postoperative increase in soluble interleukin-2 receptor serum levels as predictor for early recurrence in non-small cell lung carcinoma. *Cancer* 1992; **69**: 2458–2462.
97. Hsu MM, Ko JY, Chang YL. Elevated levels of soluble interleukin 2 receptor and tumor necrosis factor in nasopharyngeal carcinoma. *Arch Otolaryngol Head Neck Surg* 1991; **117**: 1257–1259.
98. Zielinski CC, Müller C, Tichatschek E, Aiginger P. Decreased production of soluble interleukin 2 receptor by phytohaemagglutin-stimulated peripheral blood mononuclear cells in patients with breast cancer after adjuvant therapy. *Br J Cancer* 1989; **60**: 712–714.
99. Lissoni P, Barni S, Rovelli F. The biological significance of soluble interleukin-2 receptors in solid tumors. *Eur J Cancer* 1990; **26**: 33–36.
100. Lotze MT, Custer MC, Sharrow SO, Rubin LA, Nelson DL, Rosenberg SA. *In vivo* administration of purified human interleukin-2 to patients with cancer: development of interleukin-2 receptor positive cells and circulating soluble interleukin-2 receptors following interleukin-2 administration. *Cancer Res* 1987; **47**: 2188–2195.
101. Hänninen EL, Körfer A, Hadam M, *et al.* Biological monitoring of low-dose interleukin 2 in humans: soluble interleukin 2 receptors, cytokines, and cell surface phenotypes. *Cancer Res* 1991; **50**: 6312–6316.
102. Manoussakis MN, Papadopoulos GK, Drosos AA, Moutsopoulos HM. Soluble interleukin 2 receptor molecules in the serum of patients with autoimmune diseases. *Clin Immunol Immunopathol* 1989; **50**: 321–332.
103. Wolf RE, Brelsford WG. Soluble IL-2 receptor in systemic lupus erythematosus. *Arthritis Rheum* 1988; **31**: 729–735.
104. Huang YP, Perrin LH, Miescher PA, Zubler RH. Correlation of T and B cell activities *in vitro* and serum IL-2 levels in systemic lupus erythematosus. *J Immunol* 1988; **141**: 827–833.
105. Tokano Y, Murashima A, Takasaki Y, Hashimoto H, Okomura K, Mirose S. Relation between soluble IL-2 receptor and clinical findings in patients with systemic lupus erythematosus. *Ann Rheum Dis* 1989; **48**: 803–809.
106. Ward MM, Dooley MA, Christenson VD, Pisetsky DS. The relationship between soluble interleukin-2 receptor levels and antidouble stranded DNA antibody levels in patients with systemic lupus erythematosus. *J Rheum* 1991; **18**: 235–240.
107. Wong KL, Wong RPO. Serum soluble interleukin-2 receptor in systemic lupus erythematosus: effects of disease activity and infection. *Ann Rheum Dis* 1991; **50**: 706–709.
108. Raziuddin S, Al-Janadi MA, Al-Wabel A. Soluble interleukin-2 receptor levels in serum and its relationship to T cell abnormality and clinical manifestation of the disease in patients with systemic lupus erythematosus. *J Rheum* 1991; **18**: 831–836.
109. Symons JR, Wood NC, Di Giovine FS, Duff GW. Soluble IL-2 receptor in rheumatoid arthritis. Correlation with disease activity, IL-1 and IL-2 inhibition. *J Immunol* 1988; **141**: 2612–2618.
110. Keystone EC, Snow KM, Bombardier C, Chang CH, Nelson DL, Rubin LA. Elevated soluble IL-2 receptor levels in the sera and synovial fluids of patients with rheumatoid arthritis. *Arthritis Rheum* 1988; **31**: 844–849.
111. Campen DH, Horwitz DA, Quismoria FP Jr, Ehresmann GR, Martin WJ. Serum levels of IL-2 receptor and activity of rheumatic diseases characterized by immune system activation. *Arthritis Rheum* 1988; **31**: 1358–1364.
112. Wood NC, Symons JA, Duff GW. Serum interleukin-2 receptor in rheumatoid arthritis: a prognostic indicator of disease activity? *J Autoimmunity* 1988; **1**: 353–360.
113. Rubin LA, Snow KN, Nelson DL, Keystone EC. Serum levels of soluble IL-2 receptor in the peripheral blood of patients with rheumatoid arthritis correlate with disease activity. *J Rheum* 1990; **17**: 597–602.
114. Symons JA, Wood NC, Di Giovine FS, Duff GW. Soluble CD8 in patients with rheumatic diseases. *Clin Exp Immunol* 1990; **80**: 354–359.
115. Zielinski CC, Pesau B, Müller CH. Soluble interleukin-2 receptor and soluble CD8 antigen in active rheumatoid arthritis. *Clin Immunol Immunopathol* 1990; **57**: 74–82.
116. Symons JA, McCulloch N, Wood NC, Duff GW. Soluble CD4 in patients with rheumatoid arthritis and osteoarthritis. *Clin Immunol Immunopathol* 1991; **60**: 72–82.
117. Silverman ED, Laxer RM, Nelson DL, Rubin LA. Soluble interleukin-2 receptor in juvenile rheumatoid arthritis. *J Rheum* 1991; **18**: 1398–1402.
118. Miller FW, Love LA, Turitty SA, Plotz PH. Soluble CD8 and interleukin-2 receptors are measures of disease activity in the idiopathic inflammatory myopathy. *Arthritis Rheum* 1989; **33**: S33.
119. Wolf RE, Baethge BA. Cytokines and interleukin-2 receptors in polymyositis. *Arthritis Rheum* 1989; **32**: S31.

120. Cohen-Kaminsky S, Jacques Y, Aime C, Safer D, Morel E, Berrih-Aknin S. Follow-up of soluble interleukin-2 receptor levels after thymectomy in patients with Myasthenia gravis. *Clin Immunol Immunopathol* 1992; **62**: 190–198.
121. Giordano C, Galluzzo A, Marco A, *et al.* Increased interleukin-2 receptor levels in the sera of type diabetic patients. *Diabetes Res* 1988; **8**: 135–138.
122. Giordano C, Pantò F, Caruso C, *et al.* Interleukin 2 and soluble interleukin-2-receptor secretion defect *in vitro* in newly diagnosed type I diabetic patients. *Diabetes* 1989; **38**: 310–315.
123. Lai KN, Leung JCK, Chow CC, Cockram CS. T lymphocyte activation in euthyroid Graves' ophthalmopathy: soluble interleukin 2 receptor release, cellular interleukin 2 receptor expression and interleukin 2 production. *Acta Endocrinol* 1989; **120**: 602–609.
124. Balazs C, Farid NR. Soluble interleukin-2 receptor in sera of patients with Graves' disease. *J Autoimmunol* 1991; **4**: 681–688.
125. Nakanishi K, Taniguchi Y, Otha Y. Increased soluble interleukin 2 receptor levels in autoimmune thyroid disease. *Acta Endocrinol* 1991; **125**: 253–258.
126. Mariotti S, Caturegli P, Barbesino G, Del Prete GF, Chiovato L, Pinchera A. Circulating soluble interleukin 2 receptor concentration is increased in both immunogenic and nonimmunogenic hyperthyroidism. *J Endocrinol Invest* 1991; **14**: 777–781.
127. Greenberg SJ, Marcon L, Hurwitz BJ, Waldmann TA, Nelson DL. Elevated levels of soluble interleukin-2 receptors in multiple sclerosis. *N Engl J Med* 1988; **319**: 1019–1020.
128. Trotter JL, Clifford DB, Anderson CB, van der Veen RC, Hicks BC, Banks G. Elevated serum interleukin-2 levels in chronic progressive multiple sclerosis. *N Engl J Med* 1988; **318**: 1206–1208.
129. Adachi K, Kumamoto T, Araki S. Interleukin-2 receptor levels indicate relapse in multiple sclerosis. *Lancet* 1989; **i**: 559–560.
130. Bansil S, Troiano R, Cook SD, Rohowsky-Kochan C. Serum soluble interleukin-2 receptor levels in chronic progressive, stable and steroid-created multiple sclerosis. *Acta Neurol Scand* 1991; **84**: 282–285.
131. Proujansky R, Carpenter AB. Soluble interleukin-2 receptor as a marker of lymphocyte activation in childhood Crohn's disease. *J Pediatr Gastroenterol Nutr* 1991; **13**: 277–284.
132. Brynskov J, Tvede N, Andersen CB, Vilien M. Increased concentration of interleukin 1$\beta$, Interleukin-2, and soluble interleukin-2 receptors in endoscopical mucosal biopsy specimens with active inflammatory bowel disease. *Gut* 1992; **33**: 55–58.
133. Matsura T, West GA, Klein JS, Ferraris L, Fiocchi C. Soluble interleukin-2 and CD8 and CD4 receptors in inflammatory bowel disease. *Gastroenterology* 1992; **102**: 2006–2014.
134. Schreiber S, Raedler A, Conn AR, Rombeau JL, MacDermott RP. Increased *in vitro* release of soluble interleukin-2 receptor by colonic lamina propria mononuclear cells in inflammatory bowel disease. *Gut* 1992; **33**: 236–241.
135. Williams AJK, Symons JA, Watchet K, Duff GW. Soluble interleukin-2 receptor and disease activity in Crohn's disease. *J Autoimmunity* 1992; **5**: 251–259.
136. Crabtree JE, Heatley RV, Juby LD, Howdle PD, Losowsky MS. Serum interleukin-2-receptor in coeliac disease: response to treatment and gluten challenge. *Clin Exp Immunol* 1989; **77**: 345–348.
137. Semenzato G, Cipriani A, Trentin L, *et al.* High serum levels of soluble interleukin-2 receptors in sarcoidosis. *Sarcoidosis* 1987; **4**: 25–27.
138. Laurence EC, Brosseau KQ, Berger MD, Kurman CC, Marcon L, Nelson DL. Elevated concentration of soluble interleukin-2 receptors in serum samples of bronchoalveolar lavage fluid in active sarcoidosis. *Am Rev Resp Dis* 1988; **337**: 759–764.
139. Parera M, Rivera F, Egido J, Campos A. The role of interleukin-2 (IL-2) and serum soluble IL-2 receptor cells in idiopathic IgA nephropathy. *Clin Immunol Immunopathol* 1992; **63**: 196–199.
140. Kapp A, Neuner P, Krutmann J, Luger TA, Schöpf E. Production of interleukin-2 by mononuclear cells *in vitro* in patients with atopic dermatitis and psoriasis. *Acta Dermatol Venereol* 1991; **71**: 403–406.
141. Matsumoto T, Miike T, Yamaguchi K, Murakami M, Kawabe T, Yodoi J. Serum levels of soluble IL-2 receptor, IL-4 and IgE-binding factors in childhood allergic diseases. *Clin Exp Immunol* 1991; **85**: 288–292.
142. Engel EE, Charley M, Steen VD, Medsger TA. Soluble interleukin-2 receptors in diffuse cutaneous systemic sclerosis. *Arthritis Rheum* 1989; **32**: S42.
143. Degiannis D, Siebold JR, Czarnecki M, Raskova J, Rasha K. Soluble IL-2 receptors in patients with systemic sclerosis: clinical and laboratory correlation. *Arthritis Rheum* 1990; **33**: 375–380.
144. Famularo G, Procopio A, Giacomelli R, *et al.* Soluble interleukin-2 receptor, interleukin-2 and interleukin-4 in sera and supernatants from patients with progressive systemic sclerosis. *Clin Exp Immunol* 1990; **81**: 368–372.
145. Lang BA, Silverman ED, Laxer RM, Rose V, Nelson DL, Rubin LA. Serum soluble IL-2 receptor levels in Kawasaki disease. *J Paediatr* 1990; **116**: 592–596.
146. Modica MA, Di Lorenzo G, Galluzzo A, *et al.* Soluble interleukin-2 receptor secretion defect *in vitro* in HLA-B8,DR3 positive subjects. *Autoimmunity* 1990; **7**: 87–96.
147. Caruso C, Di Lorenzo G, Modica MA, *et al.* Soluble interleukin-2 receptor release defect *in vitro* in elderly subjects. *Mech Age Develop* 1990; **59**: 27–35.
148. Ward MM, Hall RP, Pisetsky DS. Serum interleukin-2 receptor responses to immunization. *Clin Immunol Immunopathol* 1990; **57**: 120–124.
149. Tomkinson BE, Wagner DK, Nelson DL, Sullivan JL. Activated lymphocytes during acute Epstein–Barr virus infection. *J Immunol* 1987; **139**: 3802–3807.
150. Griffin DE, Ward BJ, Jauregui E, Johnson RT, Vaisberg A. Immune activation in measles. *N Engl J Med* 1989; **320**: 1667–1672.
151. Yamaguchi S, Onji M, Ohta Y. Increased serum soluble interleukin-2 receptor levels in patients with viral liver disease. *Hepatogastroenterology* 1988; **35**: 245–248.
152. Chu CM, Liaw YF. Serum levels of soluble Tac peptide in acute and chronic hepatitis B virus infection. *Clin Immunol Immunopathol* 1989; **53**: 52–58.
153. Leung NW, Leung JC, Tam JS, Lau JT, Lai KN. Effects of alpha-interferon and prednisone on serum soluble interleukin-2 receptor (sIL-2R) in chronic hepatitis B infection. *Am J Gastroenterol* 1992; **87**: 113–117.
154. Sehti KK, Naher H. Elevated titres of cell-free IL-2 receptor in serum and cerebrospinal fluid specimens of patients with acquired immunodeficiency syndrome. *Immunol Lett* 1986; **13**: 179–184.
155. Prince HE, Kleiman S, Williams AE. Soluble IL-2 receptor level in serum from blood donors seropositive for HIV. *J Immunol* 1988; **144**: 1139–1141.
156. Prince HE, Kleinman SH, Maino VC, Jackson AL. *In vitro* activation of T lymphocytes from HIV serum positive blood donors. I. Soluble interleukin-2 receptor (IL-2R) production parallels cellular IL-2R expression and DNA synthesis. *J Clin Immunol* 1988; **8**: 114–120.
157. Reddy MM, Grieco MH. Elevated soluble interleukin-2 receptor levels in serum of human immunodeficiency virus infected populations. *AIDS Res Humana Retroviruses* 1988; **4**: 115–120.
158. Imagawa DT, Lee MH, Wolinsky SM, *et al.* Human immunodeficiency virus type 1 infection in homosexual men who remain seronegative for prolonged period. *N Engl J Med* 1989; **320**: 1458–1462.
159. Honda M, Kitamura K, Matsuda K, *et al.* Soluble IL-2 receptor in AIDS. Correlation of its serum level with classification of HIV-induced diseases and its characterization. *J Immunol* 1989; **142**: 4248–4255.
160. Fahey JL, Taylor JM, Detels R, *et al.* The prognostic value of cellular and serologic markers in infection with human immunodeficiency virus type 1. *N Engl J Med* 1990; **322**: 166–172.
161. Galli M, Ridolfo AL, Balotta C, *et al.* Soluble interleukin-2 receptor decrease in the sera of HIV-infected patients treated with zidovudine. *AIDS* 1991; **5**: 1231–1235.
162. Simmonds P, Beatson D, Cuthbert RJC, *et al.* Determinants of HIV disease progression: six-year longitudinal study in the Edinburgh haemophilia/HIV cohort. *Lancet* 1991; **338**: 1159–1163.
163. Hofmann B, Nishanian P, Fahey JL, *et al.* Serum increases and lymphoid cell surface losses of IL-2 receptor CD25 in HIV infection: distinctive parameters of HIV-induced change. *Clin Immunol Immunopathol* 1991; **61**: 212–224.
164. Noronha IL, Daniel V, Schimpf K, Opelz G. Soluble interleukin-2 receptor and tumour necrosis factor-$\alpha$ in plasma of haemophilia patients infected with HIV. *Clin Exp Immunol* 1992; **87**: 287–292.
165. Deloron P, Depers JP, Coulanges P. Evolution of the levels of soluble interleukin-2 receptors during plasmodium falciparum and P. vivax malaria. *J Clin Microbiol* 1989; **27**: 1887–1889.
166. Bresson-Hadni S, Monnot-Jacquard B, Racadot E, Lenys D, Miguet JP, Vuitton DA. Soluble IL-2-receptor and CD8 in the serum and the periparasitic granuloma of patients with alveolar echinococcosis. *Eur Cytokine Netw* 1991; **2**: 339–344.
167. Vitale G, Reina G, Mansueto S, *et al.* The significance of serum soluble IL-2 receptor as a marker for active visceral leishmaniasis in Sicilian patients. *Clin Exp Immunol* 1992; **90**: 219–222.
168. Abu-Zeid YA, Theander TG, Abdulhadi NH, *et al.* Modulation of the cellular immune response during plasmodium falciparum infections in sickle cell trait individuals. *Clin Exp Immunol* 1992; **88**: 112–118.
169. Tung SK, Umland E, Matziner P, *et al.* Soluble serum IL-2 receptor levels in leprosy patients. *Clin Exp Immunol* 1987; **68**: 10–15.
170. Filley E, Andreoli A, Steele J, *et al.* A transient rise in agalactosyl IgG correlating with free interleukin 2 receptors, during episodes of erythema nodosum leprosum. *Clin Exp Immunol* 1989; **76**: 343–347.
171. Brown AE, Rieder KT, Webster HK. Prolonged elevations of soluble interleukin-2 receptors in tuberculosis. *Am Rev Resp Dis* 1989; **139**: 1036–1038.
172. Carlson IH. New markers in serum for lymphocyte activation for predicting allograft rejection. *Clin Lab Med* 1992; **12**: 99–111.
173. Colvin RB, Fuller TC, Mackeen L, Kung PC, Ip SH, Cosimi AB. Plasma interleukin 2 receptor levels in renal allograft recipients. *Clin Immunol Immunopathol* 1987; **43**: 273–276.
174. Cornaby A, Simpson MA, Rice RV, Dempsey RA, Madras PN, Monaco AP. Interleukin-2 production in plasma, urine and plasma interleukin-2 receptor levels and urine cytology as a means of monitoring renal allograft recipients. *Transplant Proc* 1988; **20**: 108–110.
175. Forsythe JLR, Shenton BK, Parrott NR, Taylor RMR, Proud G. Plasma interleukin 2 receptor levels in renal allograft dysfunction. *Transplantation* 1989; **48**: 155–157.
176. Colvin RB, Preffer FI, Fuller TC, Brown MC, Ip SH, Kung PC, Cosimi AB. A critical analysis of serum and urine interleukin-2 receptor assays in renal allograft recipients. *Transplantation* 1989; **48**: 800–804.
177. Schroeder TJ, Helling T, McKenna RM, *et al.* A multicenter study to evaluate a novel assay for quantitation of soluble interleukin 2 receptor in renal transplant recipients. *Transplantation* 1992; **53**: 34–40.
178. Perkins JD, Nelson DL, Rakela J, Grambsch PM, Krom RAF. Soluble interleukin-2 receptor level an indicator of liver allograft rejection. *Transplantation* 1989; **47**: 77–81.
179. Adams DH, Wang L, Hubscher SG, Elias E, Neuberger JM. Soluble interleukin-2 receptors in serum and bile of liver transplant recipients. *Lancet* 1989; **1**: 469–471.
180. Perkins JD, Nelson DL, Rakela J, Grambsch PM, Krom RAF. Soluble interleukin-2 receptor level as an indicator of liver allograft rejection. *Transplantation* 1989; **47**: 77–81.
181. Keshavjee SH, McRitchie DI, Rubin LA, Maurer J, Girotti M, Patterson GA. The role of soluble interleukin-2 receptor levels in the diagnosis of rejection and infection in lung transplant recipients. *Clin Invest Med* 1989; **12**: B101.
182. Young JB, Lloyd KS, Windsor NT, Cocanougher B, Lawrence EC. Relationship of elevated soluble interleukin-2 receptor level early after heart transplant to long term survival and coronary arteriopathy. *J Heart Transplant* 1989; **9**: 80–85.
183. Jutte NHPM, Hesse CJ, Balk AHMM, Mochtar B, Weimar W. Sequential measurements of soluble interleukin 2 receptor levels in plasma of heart transplant recipients. *Transplantation* 1990; **50**: 328–330.
184. Young JB, Windsor NT, Smart FW, *et al.* Inability of isolated soluble interleukin-2 receptor levels to predict biopsy rejection scores after heart transplantation. *Transplantation* 1991; **51**: 636–641.
185. Teodorczyk-Injeyan JA, Sparkes BG, Mills GB, Falk RE, Peters WJ. Increase of serum interleukin-2 receptor in thermally-injured patients. *Clin Immunol Immunopathol* 1989; **17**: 205–215.
186. Teodorczyk-Injeyan JA, Sparkes BG, Mills GB, Peters WJ. Soluble interleukin-2 receptor alpha secretion is related to altered interleukin-2 production in thermally-injured patients. *Burns* 1991; **17**: 290–295.
187. North ME, Spickett GP, Webster DB, Farrant J. Raised serum levels of CD8, CD25 and $\beta_2$-microglobulin in common variable immunodeficiency. *Clin Exp Immunol* 1991; **86**: 252–255.
188. Hory B, Racadot E, Saint-Hillier Y, Peters A, Perol C. Soluble interleukin-2 receptors in chronic renal failure. *Am J Nephrol* 1991; **11**: 276–280.
189. Wagner F, Assemi C, Lersch C, Hart R, Classen M. Soluble interleukin-2 receptor and soluble CD8 in liver cirrhosis and obstructive jaundice. *Clin Exp Immunol* 1990; **82**: 344–349.
190. Hofmann B, Bass H, Nishanian P, *et al.* Different lymphoid cell populations produce varied levels of neopterin, $\beta_2$-microglobulin and soluble IL-2 receptor when stimulated with IL-2, interferon-gamma or tumour necrosis factor-alpha. *Clin Exp Immuol* 1992; **88**: 548–554.
191. Wiebke EA, Rosenberg SA, Lotze MT. Acute immunologic effects of interleukin-2 therapy in cancer patients: decreased delayed type hypersensitivity response and decreased proliferative response to soluble antigens. *J Clin Oncol* 1988; **6**: 1440–1449.
192. Modica MA, Zambito AM, Candore G, Caruso C. Markers of T lymphocyte activation in HLA-B8,DR3 positive individuals. *Immunobiol* 1990; **181**: 257–266.
193. Candore G, Di Lorenzo G, Caruso C, *et al.* The effect of age on mitogen responsive T cell precursors in human beings is completely restored by interleukin-2. *Mech Ageing Dev* 1992; **63**: 297–307.
194. Modica MA, Colucci AT, Candore G, Caruso C. The HLA-B8,DR3 haplotype and immune response in healthy subjects. *Immunol Infect Dis* (submitted).

ACKNOWLEDGEMENTS. This work was supported by a grant of Ministero dell'Universita' e della Ricerca Scientifica e Tecnologica (60%) to Professor Calogero Caruso.

**Received 29 November 1992; accepted 15 December 1992**
